# Multi-omics integration reveals molecular networks and regulators of psoriasis

**DOI:** 10.1186/s12918-018-0671-x

**Published:** 2019-01-14

**Authors:** Yuqi Zhao, Deepali Jhamb, Le Shu, Douglas Arneson, Deepak K. Rajpal, Xia Yang

**Affiliations:** 1 Department of Integrative Biology and Physiology, University of California, Los Angeles, 610 Charles E. Young Dr. East, Los Angeles, CA 90095 USA; 2 Target Sciences, Computational Biology (US) GSK, 1250 South Collegeville Road, Collegeville, PA 19426 USA; 3 Institute for Quantitative and Computational Biosciences, University of California , 610 Charles E. Young Dr. East, Los Angeles, CA 90095 USA; 4 Molecular Biology Institute, University of California, 610 Charles E. Young Dr. East, Los Angeles, CA 90095 USA; 5 Bioinformatics Interdepartmental Program, University of California, 10 Charles E. Young Dr. East, Los Angeles, CA 90095 USA

**Keywords:** Psoriasis, GWAS, EWAS, Systems biology, Integrative genomics

## Abstract

**Background:**

Psoriasis is a complex multi-factorial disease, involving both genetic susceptibilities and environmental triggers. Genome-wide association studies (GWAS) and epigenome-wide association studies (EWAS) have been carried out to identify genetic and epigenetic variants that are associated with psoriasis. However, these loci cannot fully explain the disease pathogenesis.

**Methods:**

To achieve a comprehensive mechanistic understanding of psoriasis, we conducted a systems biology study, integrating multi-omics datasets including GWAS, EWAS, tissue-specific transcriptome, expression quantitative trait loci (eQTLs), gene networks, and biological pathways to identify the key genes, processes, and networks that are genetically and epigenetically associated with psoriasis risk.

**Results:**

This integrative genomics study identified both well-characterized (e.g., the IL17 pathway in both GWAS and EWAS) and novel biological processes (e.g., the branched chain amino acid catabolism process in GWAS and the platelet and coagulation pathway in EWAS) involved in psoriasis. Finally, by utilizing tissue-specific gene regulatory networks, we unraveled the interactions among the psoriasis-associated genes and pathways in a tissue-specific manner and detected potential key regulatory genes in the psoriasis networks.

**Conclusions:**

The integration and convergence of multi-omics signals provide deeper and comprehensive insights into the biological mechanisms associated with psoriasis susceptibility.

**Electronic supplementary material:**

The online version of this article (10.1186/s12918-018-0671-x) contains supplementary material, which is available to authorized users.

## Background

Psoriasis is a common and chronic skin disease with poorly understood etiology. It is mainly characterized by vascular remodeling, epidermal hyper-proliferation, and inflammation [1]. The development of psoriasis involves both genetic susceptibilities and environmental triggers [2]. From the genetic perspective, the heritability of psoriasis has been estimated to be 60–75% based on a population-based twin study [3]. Recent genome-wide association studies (GWAS) have identified ~ 50 genetic loci at genome-wide significance (*p* < 5e-8), together explaining ~ 22% of the genetic heritability [4, 5] and leaving two-thirds of the heritability to be further explored. In addition to genetic factors, environmental factors such as psychological stress, injuries, cigarette smoking, obesity, infections, and alcohol consumption, also play an important role in psoriasis pathogenesis. To tackle the molecular underpinnings of environmental factors, epigenome-wide association studies (EWAS) have emerged and unraveled a role for differential DNA methylation in the complex interplay between genes and the environment during disease development. For instance, Lu et al. characterized differential DNA methylation between involved and uninvolved skin lesions from patients with psoriasis and found aberrant methylation patterns in genes involved in the immune system, cell cycle and apoptosis [2].

Conventional GWAS and EWAS examine individual genetic and epigenetic markers one at a time and typically only reveal a small number of top signals due to severe multiple testing penalty. As such, they are not adequately powered to identify genes and loci with moderate to subtle effect sizes that are part of the missing heritability [6], nor are they designed to investigate tissue-specific gene-gene interactions that are increasingly recognized to play critical roles in complex disease development [7–9]. In addition, GWAS and EWAS of psoriasis have not been comprehensively examined for inter-connections or compared for commonalities and differences in the mechanistic insights inferred.

**Fig. 1:**
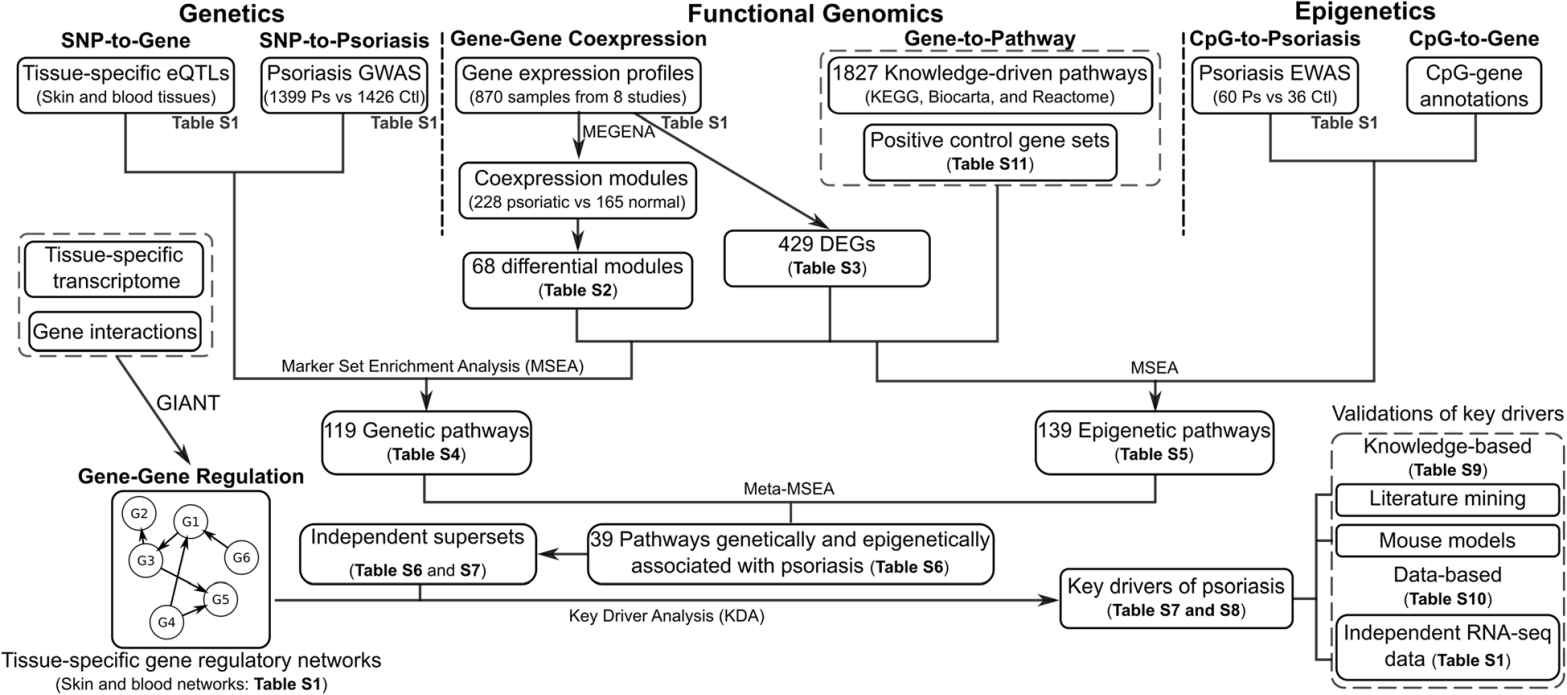
Flowchart of the study. The integrative genomic approach leverages multiple genetic and genomic datasets to uncover the mechanisms of psoriasis. The data types included are psoriasis GWAS, EWAS, gene expression profiles of human psoriatic and normal skins (Additional file 1: Table S1), tissue-specific eQTLs from skin and blood, gene regulatory networks from skin and blood, and biological pathways. The framework can be roughly divided into five steps. First, we constructed data-driven co-expression networks and curated knowledge-driven pathways. These serve as gene sets containing genes with functional relevance and relationships. Second, GWAS and EWAS of psoriasis were integrated with the gene sets using Marker Set Enrichment Analysis (MSEA) to identify genetically (via GWAS) and epigenetically (via EWAS) perturbed pathways. Third, we identified the converging psoriasis pathways from both GWAS and EWAS and merged them into independent supersets. Fourth, Bayesian gene regulatory networks were integrated with the psoriasis-associated supersets to determine key driver (KD) genes based on network topology. Finally, the KD genes and their subnetworks were cross-validated using multiple in silico methods. GIANT: Genome-scale Integrated Analysis of Networks in Tissues, the experimental details of the GIANT interface can be found in [25]

We and others have recently demonstrated that integration of multidimensional genomic resources into tissue-specific network models can improve our understanding of human complex diseases [7–12]. In this study, we apply such an integrative genomics strategy (Fig. 1) that leverages the entire summary-level data from psoriasis GWAS and EWAS (not just the genome-wide significant signals), gene expression profiles of human psoriatic and normal skins, and functional genomics information including tissue-specific expression quantitative loci (eQTLs, which reveal genetic regulation of gene expression), gene regulatory networks from key tissues involved in psoriasis, and biological pathways. Specifically, we used Mergeomics [13], a versatile and robust computational pipeline that aggregate statistical patterns of univariate associations of diverse data types and molecular networks to identify important pathways and key drivers in biological systems. Importantly, Mergeomics does not require the multi-omics datasets to be derived from the same study population, and can overcome heterogeneity between independent datasets from different studies to extract robust biological signals across data types, studies, diseases, and species. The pipeline has been successfully applied to lipid metabolism [13], diabetes [12], coronary artery disease [9, 12], and nonalcoholic fatty liver disease [11]. Extensive in vitro and in vivo validation experiments support the robustness and validity of the novel findings derived from Mergeomics [11, 12].

In this study, a comprehensive integration of tens of diverse datasets related to psoriasis using Mergeomics allowed us to unravel the gene regulatory networks that capture the full range of genetic/epigenetic perturbations (from strong to moderate and subtle), elucidate the relationships among the disease susceptibility genes/pathways informed by genetic and/or epigenetic associations, illustrate the key commonalities and differences between genetic and epigenetic mechanisms, and pinpoint key regulators for psoriasis. These findings built on multi-omics big data integration provide systems-level insights into the etiology of psoriasis and potential treatment avenues.

## Methods

We present the essential methods in the main text and the detailed descriptions can be found in Additional file 1.

### Multi-omics datasets for psoriasis

Psoriasis GWAS: Full summary statistics of psoriasis GWAS was obtained based on data accessed from dbGAP database (www.ncbi.nlm.nih.gov/gap) with accession phs000019.v1.p1 (Additional file 1: Table S1). The genotype and phenotype data was generated in 1399 psoriasis cases and 1426 controls of European ancestry [14]. No other psoriasis GWAS data with full summary statistics was publicly accessible. We used different mapping methods to link SNPs to their potential target genes (details in Additional file 1) and derived six unique sets of SNP-gene mapping: eQTL skin, eQTL blood, eQTL all (i.e., combing skin and blood eQTLs), chromosomal distance-based mapping (± 50 kb), Regulome (ENCODE-based mapping), and Combined (combing all the above methods). eQTL data sources are detailed in Additional file 1. Linkage disequilibrium (LD) between SNPs were corrected by keeping only one SNP among all SNPs in LD r2 > 0.7, a cutoff resulting in a balance between statistical power and correction of LD structure (details in Additional file 1).

Psoriasis EWAS: We searched the GEO database by keywords “psoriasis methylation” and obtained three EWAS studies for psoriasis (Additional file 1: Table S1). The raw methylation datasets were reanalyzed with the R Bioconductor packages methylumi (v2.16.0) [15] and lumi (v2.22.1) [16]. The CpG sites were mapped to adjacent genes within 5 kb [17].

Psoriasis transcriptome data: We searched the Gene Expression Omnibus (GEO) database for studies involving gene expression profiling for psoriasis. To avoid systematic bias by different experimental platforms and small sample size, we obtained the studies using the following criteria: a) the microarray studies were performed on Affymetrix Human Genome U133 platforms (GPL570/GPL571), which are the most commonly used in the psoriasis studies; b) the sample size of both the control and psoriasis groups should be over three. Applying these criteria yielded 696 gene expression profiles (Additional file 1: Table S1). We also retrieved 174 RNA sequencing samples (92 psoriatic and 82 normal skin samples) for independent validation of our main findings (under in silico validation section at the end of the Methods).

### Differentially expressed genes and differentially methylated CpG sites in psoriasis

We detected differentially expressed genes and differentially methylated CpG sites between psoriasis and normal skin using GEO2R (www.ncbi.nlm.nih.gov/geo/geo2r/), a web-based program that employs the Bioconductor packages GEOQuery [18] and limma [19] in R, with the Benjamini-Hochberg false-discovery rate (FDR) method for multiple-testing correction as its default method.

### Reconstruction of skin coexpression networks

The MEGENA (Multiscale Embedded Gene Co-expression Network Analysis) package [20] was applied to reconstruct the co-expression networks in psoriasis and normal skin separately (details in Additional file 1). The coexpression modules were compared between psoriasis and normal groups using the “Reciprocal Best Hits” method [21, 22]. Fisher’s exact test was performed to assess gene overlaps between two modules from psoriatic and normal skin networks. If the coexpression modules satisfy FDR < 0.05 in the Fisher’s exact test and were reciprocal best hits between psoriasis and normal, we define them as preserved modules. Modules failed to satisfy FDR < 0.05 were deemed as “differential modules” that were not preserved between psoriasis and normal skin. To link each coexpression module to biological processes, we used the build-in functions in the MEGENA package [20] to annotate each module with known pathways and functional categories collected from Gene Ontology, Biocarta, KEGG and Reactome databases [23, 24]. The significance of the overlap between a coexpression module and an annotation pathway was calculated using Fisher exact test with FDR < 5% to identify significant annotation terms and link the modules to biological processes.

### Knowledge-based biological pathways

The knowledge-based biological pathways included 1827 canonical pathways from the Reactome, Biocarta, and KEGG databases [23, 24] and three psoriasis related “positive control” gene sets (details in Additional file 1: Table S2).

### MSEA (marker set enrichment analysis)

To detect gene sets (knowledge-based pathways or data-driven coexpression modules) affected by molecular markers (genetic loci or methylation sites) associated with psoriasis, we used MSEA in the Mergeomics package, which has been demonstrated with superior performance compared to other gene set enrichment analysis methods [13]. For a given gene set, gene members are first mapped to markers (SNP/CpGs) based on a mapping file described above and then the disease association *p* values of the corresponding markers are extracted to test for enrichment of association signals based on a chi-squared-like test statistic, followed by FDR estimation (Additional file 1) [13]. To evaluate a gene set across multiple EWAS studies, we employed the Meta-MSEA analysis in Mergeomics, which conducts meta-analysis to retrieve robust gene sets across studies (Additional file 1).

### Merging psoriasis-associated pathways into supersets

We merged the common pathways associated with psoriasis with FDR < 5% in both GWAS and EWAS using a merging algorithm in Mergeomics [13]. Briefly, the overlap ratio r between two gene sets A and B was defined as r = (r_AB_ × r_BA_)^0.5^, where r_AB_ is the proportion of genes in A that are also in B while r_BA_ is the proportion of genes in B that are also in A. We set the overlap ratio r cutoff to > = 0.2 and also required Fisher’s exact tests for the number of shared genes among pathways to be statistically significant (FDR < 0.05). If a resulting superset had more than 500 genes, we trimmed it down to the core genes that were shared across overlapping gene sets.

### Tissue-specific gene regulatory networks and weighted key driver analysis (wKDA)

The Bayesian gene regulatory networks of skin and blood tissues were retrieved from GIANT [25]. GIANT gene networks were constructed using Bayesian modeling of tissue-specific transcriptome data from human datasets from GEO and known functional gene relationships. Using these networks, we performed wKDA [13] on the disease-associated supersets (9 common, 16 GWAS-unique, and 13 EWAS-unique supersets respectively) to determine the key drivers whose network neighbors are enriched for genes in these supersets informed by psoriasis GWAS and EWAS. The test statistic for wKDA was based on a chi-squared-like test, with FDR < 0.05 used to focus on the top robust KDs (details in Additional file 1).

### In silico validation of KDs

To investigate the relevance of the identified KDs to psoriasis based on previous literature evidence, we assessed relevant information from different resources: 1) Mouse Phenotypes from the Mouse Genome Database (MGD) [26], 2) the Gene Ontology annotations [27], 3) literature support for disease implication using Linguamatics [28], Polysearch [29], and COREMINE (http://www.coremine.com/medical).

Besides literature mining, we utilized RNA-seq transcriptomes (GEO accession: GSE54456) from 92 psoriatic and 82 normal punch biopsies [30], as independent sets of transcriptomic data to analyze the transcriptomic perturbations of KD subnetworks in lesional psoriatic skin. The transcriptomic patterns of the KD subnetworks were assessed using the Gene Set Enrichment Analysis (GSEA, the latest version gsea2–2.2.0.jar) [31], which determines whether an a priori defined set of genes shows statistically significant, concordant differences between two biological states (the psoriatic and normal skins in this case) based on the Kolmogorov-Smirnov statistic.

## Results

### Construction of skin co-expression networks in psoriasis using transcriptome data

We first analyzed potential changes in gene-gene relationships involved in psoriasis based on gene co-expression patterns. We retrieved skin transcriptomic datasets from 696 samples in eight transcriptome studies (Additional file 1: Table S1). Using a multiscale gene coexpression network modeling approach MEGENA [20], we reconstructed a psoriatic network comprised of 228 co-expression modules and a normal skin network comprised of 165 modules (Fig. 1). We identified 68 differential coexpression modules that are not preserved between the two networks, including 24 from psoriatic skins and 44 from normal skins at FDR < 0.05 (Additional file 1: Table S3; details in Methods). Modules from normal skins can be disrupted in disease conditions, and pathogenic modules may uniquely form in psoriatic skins; both types of modules can be informative for disease pathogenesis. These modules were involved in diverse biological processes, such as “IL12 pathway”, “T Cell Receptor (TCR) signaling”, and “branched chain amino acid (BCAA) catabolism” (Additional file 1: Table S3). In addition, we identified 429 differentially expressed genes (DEGs) between psoriatic and normal skins at FDR < 0.05 (Additional file 1: Table S4). The 68 differential coexpression modules and the DEG set serve as a collection of gene sets that inform on functional gene-gene connections that are potentially perturbed by genetic and epigenetic risks of psoriasis.

### Coexpression networks and pathways associated with psoriasis in GWAS

The above transcriptome-based analyses are correlative in nature and cannot differentiate the upstream, disease-causing processes from those that are downstream, reactive to the disease condition. Genetic signals from GWAS, due to their inheritable nature, precede disease development and therefore have the power to infer causality. To this end, we integrated the 68 differential coexpression modules and the DEG set from the above skin transcriptome analyses with the full statistics of a large psoriasis GWAS from the Collaborative Association Study of Psoriasis [14] using MSEA (see Methods) [13]. To complement the data-driven gene sets, we also incorporated 1827 knowledge-based pathways curated from KEGG, Biocarta, and Reactome databases as complementary sets of genes that are functionally related. Furthermore, we included three predefined psoriasis-related gene sets as positive controls based on 1) top psoriasis GWAS hits in GWAS Catalog [32], 2) the well-established IL23/IL17 pathway [33, 34], and 3) previously refined psoriasis gene signatures based on differential analysis of transcriptome data (details in Additional file 1: Table S2) [35, 36]. Briefly, single nucleotide polymorphisms (SNPs) were mapped to genes in each gene set using chromosomal location (SNPs were mapped to adjacent genes) or function-based mapping using blood and skin eQTLs and ENCODE information (SNPs with functional evidence support were mapped to adjacent genes). Skin eQTLs were chosen because of the direct relevance to psoriasis, and blood eQTLs were chosen because they mainly represent gene regulation in immune cells, which play a significant role in psoriasis. The psoriasis GWAS association *p* values for the mapped SNPs from each gene set were then extracted and compared with SNPs mapped to random gene sets of matching sizes to assess the aggregate GWAS association strength of a gene set with psoriasis (detailed in Methods).

We found 14 (out of 68; 10 from normal skin and 4 from psoriatic skin) differential coexpression modules and 105 (out of 1827) canonical pathways to be significantly enriched for genetic risk variants of psoriasis (FDR < 5%; Additional file 1: Table S5). As expected, the “positive control sets based on top GWAS” hits and the “IL23/IL17 immune pathway” were among the top signals in terms of enrichment for genetic variants (Additional file 1: Table S5). The two transcriptome-based DEG gene sets in psoriasis (both our own DEGs and those from the previous studies [35, 36]), however, were not significant, suggesting that these genes are likely not causal for psoriasis but may be downstream molecular changes. This result agrees with the conclusion from a previous study [37] that differential co-expression is more informative for causal disease mechanisms, whereas differential gene expression is more likely reflective of events downstream of diseases and may be more useful as biomarkers rather than causal targets.

**Fig. 2:**
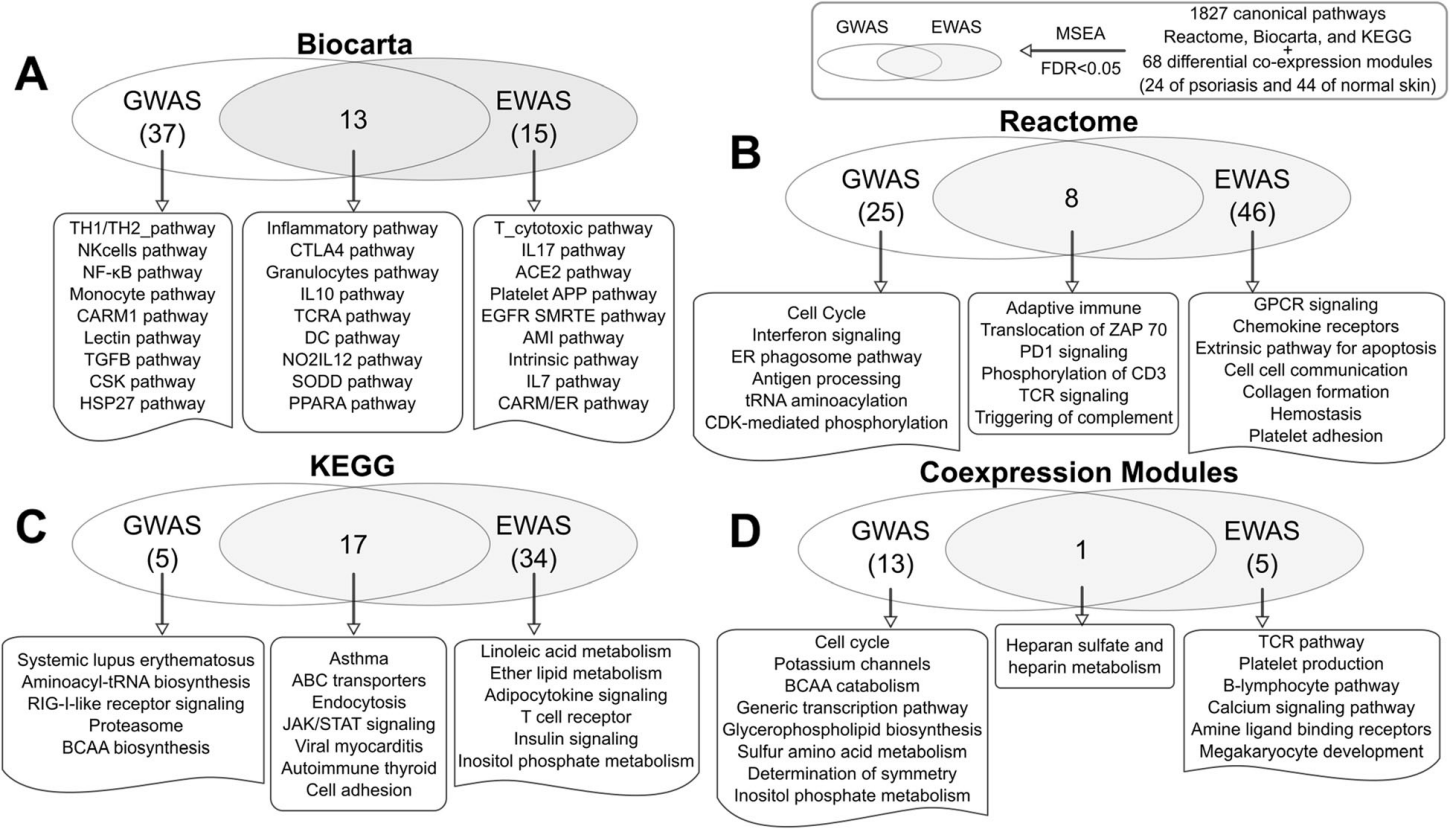
Comparison of significant pathways between GWAS and EWAS. Panels A-D represent significant canonical pathways/coexpression modules from Biocarta (**a**), Reactome (**b**), KEGG (**c**), and coexpression networks (**d**), respectively, that are associated with in psoriasis in GWAS and EWAS. The detailed MSEA results in GWAS and EWAS can be found in Additional file 1: Tables S5 and S6. The pathways are derived from various databases including Biocarta, Reactome, and KEGG, and were intersected with GWAS or EWAS using our MSEA procedure to identify pathways whose genes contain genetic or epigenetic variants showing coordinated association with psoriasis in GWAS or EWAS. “BCAA biosynthesis” stands for branched chain amino acids biosynthesis. In Additional file 1: Table S5, the corresponding full pathway name is “valine, leucine, and isoleucine biosynthesis”, where valine, leucine, and isoleucine are BCAAs

Interestingly, although we could not obtain access to the full GWAS summary statistics from the latest psoriasis GWAS which reported results from ~ 40,000 individuals from eight different Caucasian cohorts [38], our analysis using an older GWAS dataset [14] (phs000019.v1.p1 used in our analysis) involving a much smaller sample size (~ 2800 individuals) was able to capture pathways identified in the latest GWAS study of ~ 40,000 individuals, such as “lymphocyte differentiation/regulation”, “Type I interferon”, “pattern recognition and response to virus/bacteria”, and “NF-κB cascade”, serving as a cross-validation of our findings. Moreover, our approach aggregating the full spectrum of GWAS had the power to capture many more biological processes than those informed by the GWAS hits from the latest psoriasis GWAS study, such as the “cell cycle”, “ER phagosome”, “proteasome”, “BCAA biosynthesis” and “BCAA catabolism” (Fig. 2).

The incorporation of tissue-specific eQTLs to guide SNP-gene mapping allowed us to capture tissue-dependency of the pathways. For example, when blood eQTLs were used for SNP-gene mapping in the analysis, we could retrieve pathways such as “IL10”, “TH1/TH2”, and “granulocytes” pathways; when skin eQTLs were used, coexpression modules involved in “sulfur amino acid metabolism” and “cytokine signaling” were identified.

### Coexpression networks and pathways associated with psoriasis in EWAS

In addition to GWAS, epigenetic associations can also reveal causal processes that are subject to perturbations by environmental inducers. We therefore conducted MSEA using three accessible psoriasis EWAS datasets (Additional file 1: Table S1; details in Methods). We identified 133 (out of 1827) knowledge-based pathways and 6 differential coexpression modules (out of 68; 5 from normal skin and 1 from psoriatic skin) to be significantly enriched for psoriasis-associated DNA methylation signals in > = 2 EWAS studies or in a meta-analysis across the three EWAS studies (FDR < 5%; Additional file 1: Table S6). Among these, many epigenetically associated pathways reported by a recent systematic review on the epigenetics of psoriatic disease [39], such as “IL17 pathway”, “Natural killer cell-mediated cytotoxicity”, and “Leukocyte transendothelial migration” were captured in our analysis. Besides immune related processes, pathways such as lipid metabolism [40], insulin signaling, adipokine signaling, collagen formation, and cell-cell communication were also found to be enriched for psoriasis EWAS signals (Fig. 2).

### Overlap and differences in pathways and networks informed by GWAS and EWAS

We compared the pathways and network modules identified in GWAS and EWAS and found that 39 pathways overlapped (38 canonical pathways and 1 coexpression module from normal skin), suggesting that both genetic and epigenetic risks could converge on similar sets of genes and pathways (Fig. 2; Table 1; Additional file 1: Table S7). The overlapping signals between GWAS and EWAS included many inflammatory and immune response pathways. Notably, the IL17 component, a well-established psoriasis causal pathway, is affected by both GWAS and EWAS signals. In addition, “PPARA signaling”, “ABC transporters”, and “endocytosis” pathways, and a coexpression module enriched for genes involved in heparin sulfate/heparin metabolism are also among the shared signals between GWAS and EWAS.

**Table 1:** Top shared pathways associated with psoriasis identified in GWAS and EWAS at FDR < 5%, and their corresponding network key drivers (KDs) in skin and blood networks

Supersets	Module Size	Resources	Pathways	KDs in skin	KDs in blood
S1: Antigen processing and presentation	149	KEGG	Antigen processing and presentation	HLA-B, GBP1, C3, HLA-A, PSMB9	HLA-C, WARS, BCL3, BIRC3, SAT1
		KEGG	Viral myocarditis		
		KEGG	Autoimmune thyroid disease		
		KEGG	Type-I Diabetes mellitus		
		KEGG	Graft versus host disease		
		KEGG	Allograft rejection		
S2: Intestinal immune network for IGA production	45	KEGG	Asthma	N/A	BIRC3, SELL, LAPTM5, IRF8, CD40
		KEGG	Intestinal immune network for IGA production		
S3: Cytokine cytokine receptor interaction	327	KEGG	Cytokine-cytokine receptor interaction	GRB2, STAT1, TNFAIP3, TNFSF10, EGFR	BIRC3, SLA, PLAUR, LAPTM5, CSF2RB
		KEGG	JAK-STAT signaling pathway		
S4: TCR signaling	51	Reactome	TCR signaling	LCK, CHUK, NFKBIA, NFKB2, FYB	LCK, ZAP70, CD247, CD3E, CD8A
		Reactome	Generation of second messenger molecules		
		Reactome	PD1 signaling		
		Reactome	Phosphorylation of CD3 and TCR zeta chains		
		Reactome	Translocation of ZAP 70 to immunological synapse		
S5: CTLA4 pathway	31	Biocarta	CTL pathway	LCK, FYB, IKZF1, GAB1	LCK, CD247, GZMA, CD3E, NKG7
		Biocarta	CTLA4 pathway		
		Biocarta	TCRA pathway		
S6: LAIR pathway	22	Biocarta	Lair pathway	TNFAIP3, ICAM1	ITGAL, IFITM2, BIRC3, HLA-DPB1, MSN
		Biocarta	Granulocytes pathway		
S7: IL10 pathway	24	Biocarta	IL10 pathway	IL6ST, STAT1, ICAM1	STAT3, LAPTM5, BCL3, IFNGR1, ARPC1B
		Biocarta	IL22BP pathway		
S8: Inflammatory pathway	43	Biocarta	Inflammation pathway	MYD88	LAPTM5, CSF1R
		Biocarta	Cytokine pathway		
		Biocarta	DC pathway		
ABC transporters	44	KEGG	ABC transporters	N/A	N/A
Adaptive immune system	522	Reactome	Adaptive immune system	PSMD13, PSMD14, PSMC5, PSMA1, PSMD8	PSMD6, PSMC5, PSMD1, PSMC1, PSMD4
Cell adhesion molecules (CAMs)	114	KEGG	Cell adhesion molecules (CAMs)	LCK	LCK, ITGAL, SLA, LAPTM5, LILRB2
Endocytosis	177	KEGG	Endocytosis	GRB2, EGFR, RAB5A, UBC, CBL	FYN, CD58, ATP6V0D1, LHFPL2
Hematopoietic cell lineage	83	KEGG	Hematopoietic cell lineage	MCL1, TIMP1	CSF1R, LAPTM5, PLAUR, SERPINB1, IRF8
Heparan sulfate biosynthesis and metabolism	41	Coexpression	Heparan sulfate biosynthesis and metabolism	THYN1, BBS4, YBX1, HSDL1, GNL3	N/A
Immunoregulatory interactions between a lymphoid and a non-lymphoid cell	64	Reactome	Immunoregulatory interactions between a lymphoid and a non-lymphoid cell	LCK, ICAM1, HLA-F	LCK, KLRK1, CD247, LY96, PRF1
Initial triggering of complement	16	Reactome	Initial triggering of complement	N/A	IFITM2, C2, C1QB, CCL18, C1R
Leishmania infection	58	KEGG	Leishmania infection	MYD88, IER3, NFKB1, NFKBIA, TNFAIP3	PLAUR, BCL3, BIRC3, LAPTM5, CXCL2
Natural killer cell-mediated cytotoxicity	132	KEGG	Natural killer cell mediated cytotoxicity	GRB2, LCK, FYN, RAC2, ABL1	LCK, SLA, PTPN6, GZMA, PRF1
NO2IL12 pathway	17	Biocarta	NO2IL12 pathway	LCK	CD3E, GZMK, GZMA, LCK, ZAP70
PPARA pathway	58	Biocarta	PPARA pathway	AR, FOS, IER2, TRIB1, PPP1R15A	TRIB1, NFKBIA, ZFP36, BCL6, BTG2
Primary immunodeficiency	35	KEGG	Primary immunodeficiency	LCK, IL32	CD247, CD8A, LCK, CD3E, MS4A1
SODD pathway	10	Biocarta	SODD pathway	TNFSF10, CASP8, BIRC2, LYN	TRAF1, IL4R, LTB, CEBPD, FAS

Note: The detailed MSEA results in GWAS and EWAS are in Additional file 1: Tables S5 and S6. The detailed results and full list of key driver genes identified in skin and blood networks are in Additional file 1: Table S9

We also identified unique aspects of genetically vs epigenetically perturbed signals (Fig. 2). For example, the BCAA biosynthesis/catabolism processes are unique to GWAS, whereas platelet and coagulation, insulin signaling, and lipid metabolism pathways appear to be more specific to EWAS, suggesting that genetic and environmental factors also perturb different pathways leading to psoriasis.

### Merging pathways into independent supersets

We focused on the shared processes between GWAS and EWAS for the downstream analysis, as these reflect the most reproducible findings between genetic and epigenetic signals in the current study. Because we integrated the pathways and coexpression modules from diverse sources, many of the detected pathways may share gene members and can be highly overlapping. To reduce the redundancy, we merged the 39 consistent gene sets between GWAS and EWAS into 22 independent supersets by combining the overlapping ones with gene overlap ratio *r* > 0.2 and FDR < 0.05 (see methods for details) into supersets (8) while keeping the non-overlapping ones (14) intact (Table 1; Methods in Additional file 1; more detailed results in Additional file 1: Table S7). To confirm the merged supersets still carry the GWAS and EWAS information, we performed meta-analysis of the 22 supersets across the GWAS and EWAS studies separately and found 20 satisfying FDR < 5% in both meta-MSEA of GWAS and EWAS (Additional file 1: Table S7). As HLA genes (human leukocyte antigen) are located in close physical proximity on chromosomes and tend to be co-regulated, they might introduce statistical artefacts. Excluding HLA genes in the meta analyses confirmed 9 supersets at a stringent cutoff of FDR < 5.0e-4, 4 of which do not contain HLA genes (“Cytokine Cytokine Receptor Interaction”, “ABC Transporters”, “LAIR Pathway”, and “Heparan sulfate and heparin biosynthesis and metabolism”) and 5 of which contain HLA genes but retained significance even after removal of these genes (“CTLA4 Pathway”, “Intestinal Immune Network For IGA Production”, “Hematopoietic Cell Lineage”, “TCR Signaling”, and “Antigen Processing And Presentation”), suggesting that other genes in the latter category also contribute to the genetic and epigenetic signal enrichment. These were considered as a prioritized set of robustly shared processes between GWAS and EWAS of psoriasis, and we included the HLA genes in these 9 supersets for the downstream network analyses.

In addition, we merged the gene sets informed only by GWAS (82) or EWAS (100) into relatively independent supersets separately, resulting in 46 supersets from GWAS and 57 from EWAS. As these supersets may still share gene members and can be highly overlapping, we then applied Fisher’s exact test with FDR adjustment to estimate the overlaps among GWAS, EWAS, and Common supersets. If a superset from one list does not meet FDR < 0.05 threshold in enrichment analysis with any supersets from the other lists, we define them as a “unique” superset. We identified 16 and 13 unique supersets for GWAS and EWAS respectively (Additional file 1: Table S8), which are involved in diverse processes, such as “aminoacyl tRNA biosynthesis”, “platelet homeostasis”, and “sulfur amino acid metabolism” that are unique to GWAS and “neuroactive ligand receptor interaction”, “lipid metabolism”, “extracellular matrix organization” that are unique to EWAS.

### Identification and validation of central regulators for psoriasis

To explore the interactions between genes within the 9 prioritized psoriasis-associated gene sets (total 1397 genes) common to GWAS and EWAS and to detect important hub genes (termed key drivers or KDs herein), we used a weighted key driver analysis (wKDA) implemented in Mergeomics [13] and networks depicting detailed gene-gene regulatory relationships in skin and blood tissues (see Methods for details). KDs are defined as network nodes whose neighborhoods are over-represented with genes in the psoriasis supersets informed by both GWAS and EWAS. We identified 133 unique KDs satisfying FDR < 5% for the nine psoriasis supersets in the blood or skin networks (Additional file 1: Table S9).

**Fig. 3:**
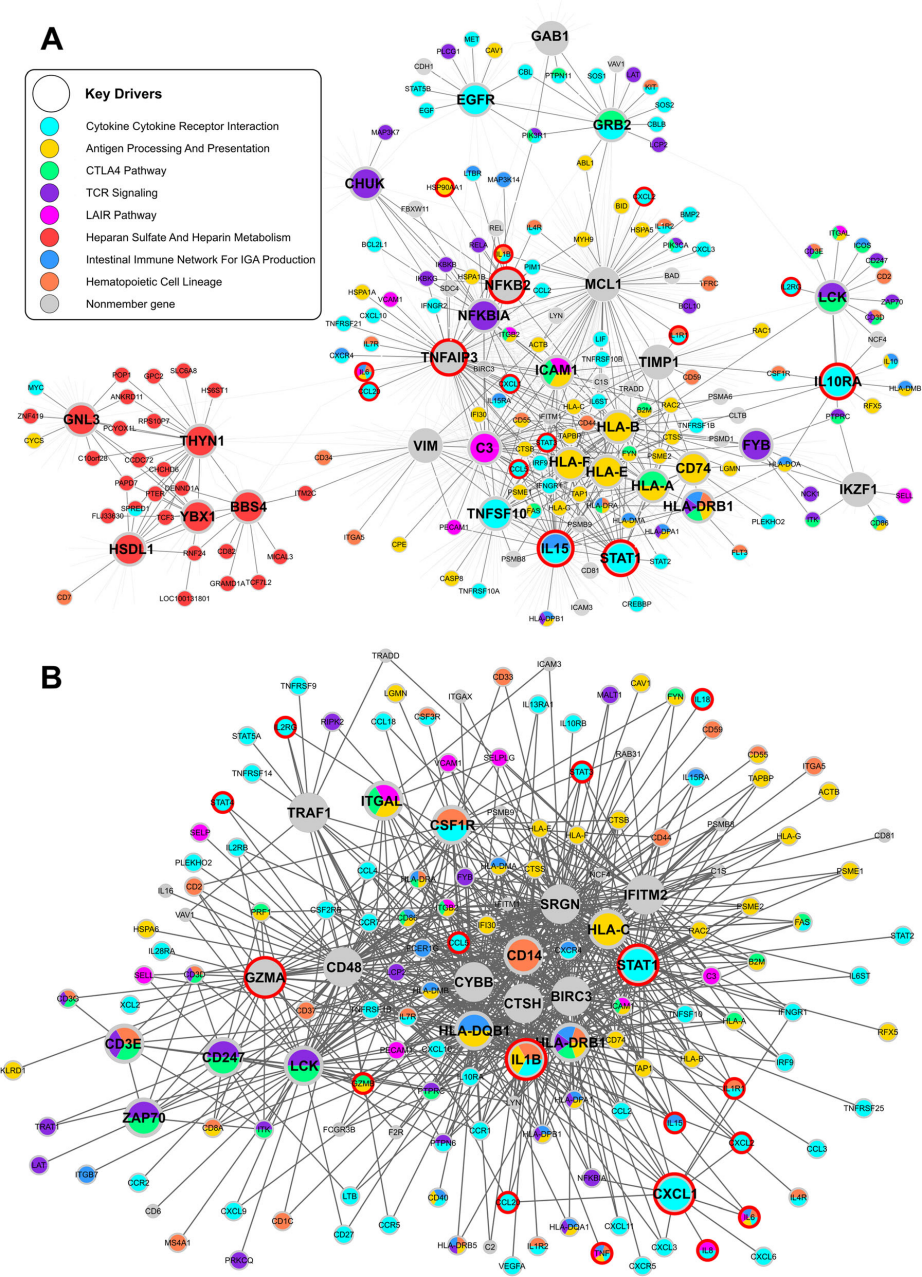
Tissue-specific gene regulatory network of the top KDs in psoriasis. Panel (**a**) and (**b**) show the first level skin (**a**) and blood (**b**) subnetworks for top KDs derived from wKDA. The genes are colored according to the common processes associated with psoriasis in both GWAS and EWAS. The bigger nodes are the top KDs. Nodes with red outlines are known genes in the IL23/IL17 immune positive control pathway

In the skin network, we identify KDs such as *ICAM1*, *IL15*, *STAT1*, *TNFAIP3*, and *GRB2* to be the network hubs connecting numerous genes in the psoriasis-associated supersets (Fig. 3a). These KD subnetworks in skin provide insights into gene regulations involved in the pathogenesis of psoriasis. For instance, the *TNFAIP3* subnetwork includes crucial factors involved in the initiation of a psoriatic skin lesion, such as *IFNGR2*, *IL1B*, *IL6*, and *CXCL10* which drive T cell-mediated inflammation and keratinocyte activation and proliferation. These later events further promote the activation of inflammatory cells such as neutrophils and macrophages to contribute to the formation of an inflamed cutaneous plaque [41]. Interestingly, the heparin metabolism pathway genes form a subnetwork surrounding KDs such as *BBS4, GNL3,* and *THYN1*, and this subnetwork is rather remote from the main skin subnetwork connected by the other KDs.

The psoriasis-associated supersets were also found to be closely linked in the blood network via KDs such as *CTSH*, *IL1B*, *STAT1*, and *IFITM2* (Fig. 3b). We also identified 36 KDs that were shared across multiple psoriasis supersets and tissue-specific regulatory networks, such as *LCK* and *STAT1* (Fig. 3). Genes with strong signals in both GWAS (*p* < 5e-8) and EWAS (*p* < 1e-5) were mostly peripheral nodes in the networks (Fig. 3), such as *C2*, *FYN*, *ICAM3*, *LTB*, *PSMB8*, *TAP1*, and *TNF*. Exclusion of HLA genes from the nine psoriasis-associated supersets did not appear to have major influence on the KDs identified (Additional file 1: Table S9). This network analysis revealed tight connections between the psoriasis-associated processes and the key regulators orchestrating the interactions.

The networks identified here also capture the relationships between drug targets for psoriasis. In the skin and blood psoriasis subnetworks, both existing (e.g., *CCL2*, *IL8*, *CD2*, *LCK*, *SELL*, *PRKCQ*, and *STAT1*) and potential drug targets (e.g., *IL4R*, *IL1B*, *CCL20*, *CCL4*, *CCR7*, *CXCL10*, *CXCL9*, *CXCR4*, *IL7R*, *LYN*, and *TNFRSF21*) [36, 41] were found to be closely connected via several KDs (*TNFAIP3*, *STAT1*, *NFKB2*, *MCL1*, *LCK*, *IL15*, *IKZF1*, and *ICAM1*) and are involved in immune system and cell migration. Besides, genes in the IL23/IL17 immune positive control pathway are over-represented in the networks (Fisher’s Exact Test *P* values < 1.0e-5 and fold changes > 5.0 for both skin and blood networks; Fig. 3).

**Fig. 4:**
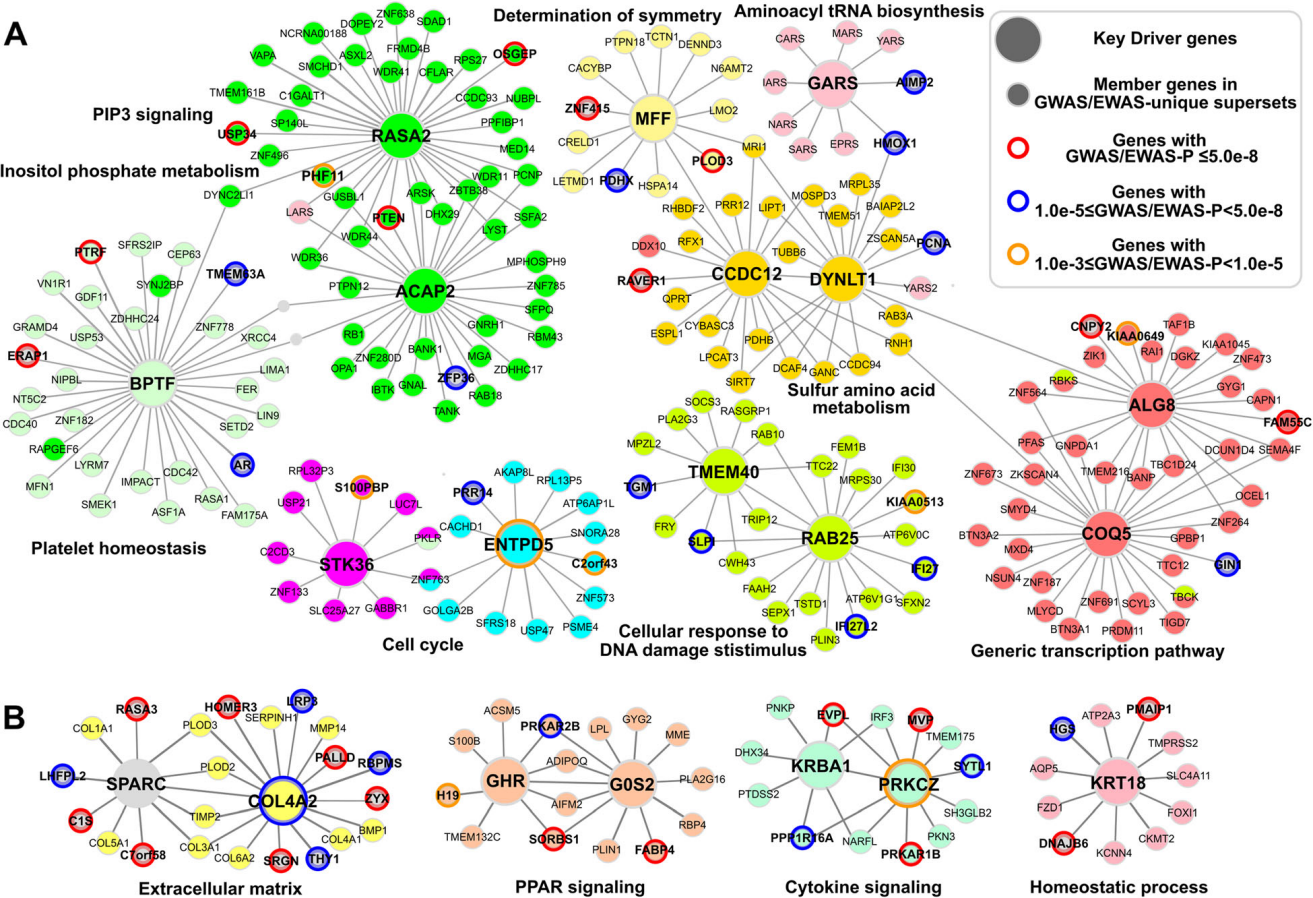
GWAS- and EWAS-unique KD subnetworks in psoriasis. Panel (**a**) and (**b**) show the GWAS- and EWAS-unique subnetworks for top KDs derived from wKDA. The genes are colored according to the unique processes associated with psoriasis in GWAS (**a**) or EWAS (**b**). The bigger nodes are the top KDs. Genes with moderate (1.0e-3 < *p* < 5.0e-8) to strong (*p* < 5.0e-8) GWAS/EWAS signals are indicated by the bold outline

We also applied KDA to the supersets unique to GWAS or EWAS and identified top KDs for these gene sets (Additional file 1: Table S8). In the skin subnetworks (Fig. 4a), the top KDs (e.g., *BPTF*, *RASA2*, *MFF*, and *ALG8*) tend to be surrounded by partner genes with moderate to strong GWAS signals (*p* values from < 1.0e-3 to < 5.0e-8). The top KD subnetworks for the EWAS-unique supersets in the skin tissue are enriched with strong EWAS signals (e.g., *SPARC* and *COL4A2* for extracellular matrix and *GHR* and *G0S2* for PPAR signaling; Fig. 4b).

### In silico validation of the KDs and their corresponding subnetworks

To cross-validate the significance of the KDs in psoriasis, we carried out a comprehensive in silico analysis of 1) the 36 KDs shared between psoriasis supersets and tissue networks for the common supersets between GWAS and EWAS, 2) 19 KDs for GWAS-unique supersets, and 3) 7 KDs for EWAS-unique supersets (see Methods for details). Briefly, we queried the KDs for evidence of psoriasis association based on the following criteria: gene knockout and mouse mutation models, psoriasis gene signatures [36], GWAS (*p* < 5.0E-8) or EWAS signals (*p* < 1.0E-5). Our search revealed that the 47 and 83% of the KDs for the common supersets are associated with psoriasis or relevant conditions in > = 2 or at least one of the above criteria, respectively; five KDs (*RAB25*, *TMEM40*, *COL4A2*, *GHR*, and *PRKCZ*) for the GWAS- or EWAS-unique supersets were associated with psoriasis or relevant conditions in at least one of the criteria (Additional file 1: Table S10).

We hypothesize that if the KD subnetworks are important for psoriasis, the expression levels of the genes in the KD subnetworks are more likely to be perturbed in psoriatic patients. To this end, we analyzed the expression profile alterations of the KD subnetworks between lesional psoriatic and normal skins using RNA-seq transcriptomes of 92 psoriatic and 82 normal skin samples (see Methods for details), which are completely independent of the other datasets used in the previous analyses. Among the 62 KD subnetworks (36 KDs from the shared supersets and 26 KDs from the GWAS/EWAS-unique supersets),10 subnetworks showed significant up-regulation patterns in psoriasis vs control at FDR < 5%), including *HLA-G*, *STAT1*, *TNFAIP3*, *BIRC3*, *TNFSF10*, *HLA-E*, *HLA-A*, *IL1B*, *LCK*, and *IL15* (Additional file 1: Table S11). Encouragingly, all of these 10 KDs were among those associated with psoriasis in the above bioinformatic validation analysis (Additional file 1: Table S10). Taken together, many of the predicted KDs exhibit evidence for involvement in psoriasis in independent studies.

## Discussion

High-throughput genomic studies have revealed a plethora of genomic and epigenomic changes contributing to psoriasis via GWAS, EWAS, and transcriptome profiling studies. However, an integrative systems analysis fully utilizing the complementarity of diverse omics data has not been conducted to capture a comprehensive view of disease regulation. To address this challenge, we integrated psoriasis GWAS, EWAS, functional genomics information (eQTLs and ENCODE), knowledge-driven pathways, transcriptome, and data-driven networks to uncover biological processes and key regulators mediating the actions of psoriasis genetic and epigenetic signals. This systematic multi-omics integration unraveled both shared and unique biological processes and gene networks associated with psoriasis between GWAS and EWAS, uncovered interactions between psoriasis genes and processes, and prioritized potential central regulators of disease pathogenesis. The comprehensive insights obtained would not have been possible without a thorough utilization and integration of the diverse existing datasets.

Many of the identified pathways replicated previous findings. For example, the IL17 pathway, one of the most well-known immune processes underlying psoriasis pathogenesis, exhibited strong enrichment for psoriasis associated genetic and epigenetic variants. Other pathways replicated based on genetic evidence include “NO2-dependent IL12 pathway”, “Th1/Th2 pathway”, and “Natural killer T cell” [6, 42]. In addition, “Cytokine and Chemokine signaling” and “JAK/STAT signaling” were replicated using psoriasis epigenetic signals [43, 44]. The retrieval of the known biology supports the validity of our analytical framework.

As our multi-omics integration leveraged the full spectrum of disease association (from strong to moderate and subtle signals) as well as functional information such as eQTLs, ENCODE, pathways, and gene networks, we observed numerous novel processes for psoriasis, such as the BCAA, ER phagosome, and proteosome pathways in GWAS and the platelet and coagulation, lipid metabolism, insulin signaling, adipokine signaling, collagen formation, and cell-cell communication pathways in EWAS (Fig. 2). The identification of multiple metabolism related pathways such as BCAA, lipid, and insulin signaling supports the observed correlation between psoriasis and metabolic disorders. The incorporation of genetic and epigenetic association information in our analysis, which informs on upstream gene regulatory events, suggests that these pathways are not merely correlated with psoriasis but likely play causal roles in disease development. For example, decreased levels of valine-leucine/isoleucine ratios were previously found in psoriatic lesions compared to non-lesional psoriatic skin [45]. In our study, the enrichment for psoriasis genetic signals in this pathway suggests its potential causal role. BCAAs are important amino acid nutrient signals that have direct and indirect effects in the regulation of metabolic processes such as glucose homeostasis, lipid metabolism, body weight, and insulin signaling, which can subsequently influence systemic inflammation [46]. In addition, immune cells oxidize BCAA as fuel sources and incorporate BCAA as the precursors for the synthesis of new immune cells, effector molecules, and protective molecules [47]. Lack of BCAAs in diet (or abnormally decreased BCAA catabolism) impairs many aspects of immune function and increases susceptibility to pathogens mainly through changes in the NF-κB and mTOR signaling pathways, subsequently increasing pro-inflammatory cytokines and decreasing anti-inflammatory cytokines (e.g., IL-10 and TGF-β1) [47]. The epigenetic connection between platelet adhesion and psoriasis is also interesting. The platelets have been shown to stimulate angiogenic vessel growth [48], which is an early pathogenic event in psoriasis [49]. A recent study showed that platelet P-selectin, functioning as a cell adhesion molecule on the surfaces of activated endothelial cells, might be used as an efficacy biomarker to monitor treatment success in psoriasis [50].

By investigating both GWAS and EWAS in the same study, we found converging pathways both genetically and epigenetically associated with psoriasis, making these a robust and prioritized set of pathways for future mechanistic and therapeutic investigations. These common pathways in GWAS and EWAS can be partitioned into 22 categories, including many previously implicated processes such as “Cytokine signaling”, “JAK/STAT signaling”, and “PPARA pathway”, and novel pathways such as “ABC transporters” and “Endocytosis”. Most identified drug transporters belong to the ATP-binding cassette family expressed in the skin and might be associated with drug-induced psoriasis [51, 52].

Beside retrieving the overlapping molecular processes informed by both GWAS and EWAS in conjunction with other functional evidence, our network modeling demonstrated that the psoriasis-associated pathways interconnect via network hub genes (Fig. 3), including both well-studied psoriasis genes involved in the immune system (*HLA-A*, *ICAM1*, *IL15*, *STAT1*, and *TNFAIP3*) and novel genes which may regulate immune processes and cell cycle (*CTSH*, *GRB2*, and *IFITM2*). Among the KDs that are known psoriasis genes, *STAT1* from IL23/IL17 pathway and cytokine-cytokine receptor interaction appears to be a KD in both the blood and skin networks. Among the novel KDs, the protein encoded by *CTSH* is a lysosomal cysteine proteinase important in the overall degradation of lysosomal proteins. Other cathepsin family members, such as *CTSS* [53], *CTSK* [54], and *CTSD* [55], have been implicated in the pathology of psoriasis. *CTSH* was surrounded by *CTSS*, *CTSD* and other critical factors involved in complement and adhesion (e.g., *C3* and *ICAM1*) in the psoriasis network (Fig. 3), suggesting that it might trigger inflammatory responses by regulating the neighbors in the network. The growth factor receptor bound protein 2, encoded by *GRB2*, plays a key role in the control of thymic positive and negative selection and enhances TCR signaling [56]. *GRB2* was suggested to induce ERBB2 signaling and trigger increased cell proliferation, survival, motility, and invasiveness [57]. *IFITM2* (interferon induced transmembrane protein 2) encodes an interferon-induced transmembrane protein that contributes to the control of cell growth through a multimeric complex involved in the transduction of anti-proliferative and homotypic adhesion signals. It is induced by IFN-γ in primary keratinocytes and plays a role in keratinocyte apoptosis in atopic dermatitis patients [58]. These potential key regulators orchestrate many known disease genes and pathways in psoriasis gene networks, and warrant further experimental investigation.

Compared to previous genomics studies of psoriasis, our study is the most comprehensive in terms of the diversity of data types included (GWAS, EWAS, transcriptome, eQTLs, ENCODE), the number of data sets, and the variety of analytical strategies. Importantly, our study utilizes the full spectrum of genetic and epigenetic association signals instead of only the top genome-wide significant hits, which offers unique power to capture the missing heritability and mechanisms. We also incorporated function-guided mapping of genetic signals to target genes using eQTLs and ENCODE data and included tissue-specific gene expression patterns. As such, in this single study we were able to uncover numerous known pathways and processes revealed through decades of psoriasis research, in addition to a number of novel processes. Additionally, our study is the first to compare GWAS and EWAS to map the convergence and divergence in the genetically and epigenetically perturbed disease processes. Moreover, our network modeling enables a bird’s eye view of the pathogenic networks and offers a prioritized list of novel regulators as potential therapeutic targets.

We acknowledge the following limitations in our study. First, we could only access one full GWAS dataset out of more than 10 published GWAS studies, highlighting the challenges in data access [59]. Encouragingly, some of our new predictions based on the only accessible GWAS dataset were confirmed using the top susceptibility loci/genes identified in the latest GWAS study, indicating that our analytical approach leveraging the full summary statistics and multiple layers of genomic information can capture and convey the essential features of psoriasis. Second, the EWAS studies included are of small sample size. Although we employed a meta-analysis to enhance statistical power and focused on the converging signals between GWAS and EWAS, it is important to validate our findings in larger EWAS when available. Third, the gene regulatory networks used in our analysis do not include other regulatory molecules such as noncoding RNAs and may miss essential key regulators that are not protein-coding [30]. Fourth, our analysis does not consider directionality of the GWAS/EWAS association, as it is not straightforward to unequivocally interpret the impact of the direction of individual variant associations on the entire pathway or network. Lastly, our variant to gene mapping mainly considers gene expression regulation, as majority of the disease genetic loci affect gene expression [60], but may miss the mapping of protein sequence variants. Along the same line, the variants used in the analysis are not necessarily the causal variants but can be tag variants in the same LD block, which may lead to mis-annotation of genes. However, we expect similar association patterns for the causal and tag variants in majority of the cases.

## Conclusions

Our comprehensive integrative genomic approach helps unveil the molecular mechanisms underlying pathogenesis of psoriasis from genetic and epigenetic aspects. In addition, we identified potential central regulators of psoriasis gene networks, which opens opportunities for future experimental testing and may aid the clinical diagnosis and treatment of psoriasis.

## Additional file

Additional file 1: **Table S1.** Transcriptome, DNA methylome, and GWAS studies included in the study. **Table S2.** Knowledge-based artificial pathways from published GWAS studies and large-scale meta-profiling of diverse collections of gene expression data sets. **Table S3.** Differential coexpression modules in psoriasis. **Table S4.** Gene signatures in the psoriasis transcriptomic study. **Table S5.** MSEA results in GWAS. **Table S6.** MSEA results in EWAS. **Table S7.** Common Pathways between GWAS and EWAS. **Table S8.** GWAS/EWAS-unique supersets and key driver analysis. **Table S9.** Key driver analysis of Common supersets in psoriasis skin and blood networks. **Table S10.** In silico mining of the key driver genes using bioinformatics tools. **Table S11.** Gene expression perturbations of key driver subnetwork in lesional skin tissue in psoriasis patients. (ZIP 358 kb)
