# Supplementary material for: Multi-omics integration reveals molecular networks and regulators of psoriasis
Source: BMC Syst Biol. 2019 Jan 14;13:8. doi: 10.1186/s12918-018-0671-x (PMC6332659; doi:10.1186/s12918-018-0671-x)
Supplement: Supplementary file 1 — Table S1. Transcriptome, DNA methylome, and GWAS studies included in the study. Table S2. Knowledge-based artificial pathways from published GWAS studies and large-scale meta-profiling of diverse collections of gene expression data sets. Table S3. Differential coexpression modules in psoriasis. Table S4. Gene signatures in the psoriasis transcriptomic study. Table S5. MSEA results in GWAS. Table S6. MSEA results in EWAS. Table S7. Common Pathways between GWAS and EWAS. Table S8. GWAS/EWAS-unique supersets and key driver analysis. Table S9. Key driver analysis of Common supersets in psoriasis skin and blood networks. Table S10. In silico mining of the key driver genes using bioinformatics tools. Table S11. Gene expression perturbations of key driver subnetwork in lesional skin tissue in psoriasis patients. (ZIP 358 kb) [file 12918_2018_671_MOESM1_ESM.zip › Supplement Text.docx]

**Supplementary Text**

**for**

**Multi-omics Integration Reveals Molecular Networks and Regulators of** **Psoriasis**

Yuqi Zhao^1^, Deepali Jhamb^2^, Shu Le^1^, Douglas Arneson^1^, Deepak K Rajpal ^2,^ *, Xia Yang^1,^*

^1^Department of Integrative Biology and Physiology, University of California, Los Angeles

^2^Target Sciences Computational Biology (US), GSK

* **Corresponding Authors:**

Xia Yang, Ph.D.

Department of Integrative Biology and Physiology

University of California, Los Angeles

610 Charles E. Young Dr. East, Los Angeles, CA 90095

Phone: 310-206-1812

Email: [xyang123@ucla.edu](mailto:xyang123@ucla.edu)

Deepak Rajpal, Ph.D.

Target Sciences, Computational Biology (US)

GSK

709 Swedeland Road, King of Prussia, PA 19406

Phone: +1 610 270 5474

Email: [deepak.k.rajpal@gsk.com](mailto:deepak.k.rajpal@gsk.com)

**Supplementary Materials and Methods**

**Reconstruction of coexpression networks**

To effectively identify functional co-regulated gene modules associated with psoriasis, the MEGENA (Multiscale Embedded Gene Co-expression Network Analysis) package [1] was used to reconstruct the co-expression networks in psoriasis and normal skin separately. The correlation among gene pairs was obtained by Pearson correlation coefficient. The minimum and maximum of the coexpression modules were set to the default values in the package, 10 and 500 respectively. In MEGENA, gene-gene similarities are then filtered by false discovery rate (FDR) to minimize the impact of false positives. FDR is computed by permuting gene expression matrix across the samples (global FDR), or by directly calculating pairwise nominal p-values by Fisher’s Z-transformation. Finally, multi-scale modules were filtered to satisfy FDR of <5%.

**Knowledge-based biological pathways**

The knowledge-based biological processes were retrieved from different resources. First, we included 1827 canonical pathways from the Reactome, Biocarta, and the Kyoto Encyclopedia of Genes and Genomes (KEGG) databases [2, 3]. Second, we constructed one positive control pathway set based on candidate genes from the GWAS Catalog [4] for psoriasis. This database is a quality controlled and manually curated collection of all published GWASs assaying at least 100,000 SNPs and all SNP-trait associations with p-values<1.0e-5. Up to August 1^st^, 2017, there were eleven GWASs of psoriasis [5-15], with 149 genetic loci (GWAS P<1.0e-5) and 119 reported unique genes. Third, since IL23/IL17 signaling plays a key role in psoriasis, we used literature knowledge [16, 17] to add an IL23/IL17 immune pathway in our overall pathway collection that was subsequently used for enrichment. Last, we collected 527 unique gene signatures of psoriasis patients through large-scale meta-profiling of diverse collections of gene expression data sets [18, 19]. The knowledge-based psoriasis gene sets were listed in **Supplementary Table S2**.

**Psoriasis GWAS**

The genotype and phenotype data in 1,399 psoriasis cases and 1,426 controls of European ancestry [9] was retrieved from dbGAP database ([www.ncbi.nlm.nih.gov/gap](http://www.ncbi.nlm.nih.gov/gap)) with accession code phs000019.v1.p1. GWAS analysis between genotype and psoriasis was conducted using a refined Quasi-Likelihood Score Test [20] and the SNP-disease association p values for 438,670 SNPs were obtained. SNPs were filtered by LD using r2<0.7 as the cutoff. The filtered SNPs, 116,944 in total, were kept for downstream pathway and network analysis.

We used three different mapping methods to link SNPs to their potential target genes: a) SNPs were mapped to adjacent genes within 50 kb, a commonly used boundary for SNP-gene mapping based on chromosomal location. If multiple genes were mapped to a SNP, all mapped genes were included in the analysis. b) SNPs were mapped to genes based on tissue-specific eQTLs, which provide functional evidence for the potential roles of the SNPs in gene expression regulation in a given tissue. We combined results from eQTL studies in human skin tissue and blood [21-31] with the eQTLs from the same tissues in the GTEx database [32]. c) We integrated functional information from the Regulome database [33], which annotates SNPs in regulatory elements in the human genome based on ENCODE studies [34]. Using the above three mapping approaches, we derived six unique sets of SNP-gene mapping. These are: eSNP skin, eSNP blood, eSNP all (i.e., combing all the tissue-specific eSNPs above), Distance (chromosomal distance-based mapping), Regulome (ENCODE-based mapping), and Combined (combing all the above methods).

**MSEA (Marker Set Enrichment Analysis)**

To detect gene sets (knowledge-based pathways or data-driven coexpression modules) affected by multidimensional molecular markers (genetic loci or methylation sites) associated with psoriasis, we used MSEA in the Mergeomics package [35], which has been developed to capture disease-associated biological processes via integration of omics-disease association and functional genomics data. The default setting of MSEA takes as input 1) summary statistics from omics association studies (e.g., GWAS, EWAS), 2) mapping between omics markers and genes, and 3) functionally defined gene sets (e.g., knowledge-driven pathways or coexpression modules). For a given gene set, gene members are first mapped to markers based on a mapping file and then the disease association p values of the corresponding markers are extracted to test for enrichment of association signals based on a chi-squared-like test statistic [36]. The test statistic is defined as$\chi=\sum_{i=1}^{n} \frac{O_{i}-E_{i}}{\sqrt{E_{i}}+\kappa}$, where n denotes the number of quantile points O and E denote the observed and expected counts of signals above each quantile point, and κ = 1 is a stability parameter. We used 10 quantile points ranging from the top 50% to the top 99.9% based on the rank of GWAS or EWAS p values. The null background was estimated by permuting gene labels to generate random gene sets matching the gene number of each pathway or co-expression module, while preserving the assignment of marker to genes. For each gene set, we generated 10000 permuted gene sets, and enrichment P-values were determined from a Gaussian distribution approximated using the enrichment statistics from the 10000 permutations. Finally, Benjami-Hochberg fasle discovery rate (FDR) was estimated across all gene sets tested for each GWAS or EWAS. Gene sets satisfying FDR< 5% were considered significant.

To evaluate a gene set across multiple EWAS studies, we employed the Meta-MSEA analysis in Mergeomics, which conducts pathway- or module-level meta-analysis to retrieve robust signals across studies. Meta-MSEA applies Stouffer’s Z score method to determine the meta-Z scores based on the p values from multiple MSEA runs, then convert them back to meta P-values. The meta-FDR was calculated using Benjamini-Hochberg method.

**Tissue-specific Gene regulatory networks and Key Driver Analysis (KDA)**

The Bayesian gene regulatory networks in skin and blood were retrieved from the GIANT Bayesian models [37]. Using the networks, we performed a weighted KDA (wKDA) [35] on the disease-associated supersets to determine the key drivers that are suitable for targeted interventions to these processes. A key driver was defined as a gene that is directionally connected to a large number of genes from a psoriasis superset, compared to the expected number for a randomly selected gene within the Bayesian network. wKDA uniquely consider the edge weight information in the form of edge consistency in the networks. Specifically, a network was first screened for suitable hub genes whose degree (number of genes connected to the hub) is in the top 25% of all network nodes. Once the hubs have been defined, the psoriasis-associated gene sets were overlaid onto the molecular network to see if a particular subnetwork was enriched for the disease genes. The edges that connect a hub to its neighbors are simplified into node strengths (strength = sum of adjacent edge weights) within the neighborhood, except for the hub itself. The test statistic for the wKDA is $\chi=\frac{O-E}{\sqrt{E}-\kappa}$, where the values O and E represent the observed and expected ratios of disease genes in a hub neighborhood. In particular,$E=\frac{N_{k}N_{p}}{N}$ is estimated based on the hub degree *N_k_*, disease gene set size *N_p_* and the order of the full network *N*, with the assumption that the weight distribution is isotropic across the network. Statistical significance of the disease-enriched hubs is estimated by permuting the gene labels in the network for 10000 times and estimating the P-value based on the null distribution. To control for multiple testing, FDR<0.05 was used to focus on the top robust KDs.

**Supplementary Tables**

**Supplementary Table S1.** **Transcriptome, DNA methylome, and GWAS studies included in the study.**

**Supplementary Table S2.** **Knowledge-based artificial pathways from published GWAS studies and large-scale meta-profiling of diverse collections of gene expression data sets.**

**Supplementary Tables S3 – S9 were provided in separate Excel sheets:**

- **Supplementary Table S3. Differential coexpression modules in psoriasis.**
- **Supplementary Table S4. Gene signatures in the psoriasis transcriptomic study.**
- **Supplementary Table S5. MSEA results in GWAS.**
- **Supplementary Table S6. MSEA results in EWAS.**
- **Supplementary Table S7. Common Pathways between GWAS and EWAS.**
- **Supplementary Table S8. GWAS/EWAS-unique supersets and key driver analysis.**
- **Supplementary Table S9. Key driver analysis of Common supersets in psoriasis skin and blood networks.**

**Supplementary Table S10. In silico mining of the key driver genes using bioinformatics tools.**

**Supplementary Table S11. Gene expression perturbations of key driver subnetwork in lesional skin tissue in psoriasis patients.**

**Supplementary Table S1.** **Omics data sets included in the study.**

| **Accessions^*^** | **Conditions** | **Sample sizes** | **Platforms** | **Usage of datasets** | **References** |
| --- | --- | --- | --- | --- | --- |
| **Transcriptome** | | | | | |
| GSE13355 | Control vs Psoriasis | 180 | Affymetrix Human Genome U133 Plus | 1. Reconstruction of coexpression networks; 2. Detection of gene signatures | [9, 38] |
| GSE14905 | Control vs Psoriasis | 82 | Affymetrix Human Genome U133 Plus |  | [39] |
| GSE30999 | Psoriatic lesional vs non-lesional skin | 170 | Affymetrix Human Genome U133 Plus |  | [40] |
| GSE34248 | Psoriatic lesional vs non-lesional skin | 28 | Affymetrix Human Genome U133 Plus |  | [41] |
| GSE41662 | Psoriatic lesional vs non-lesional skin | 48 | Affymetrix Human Genome U133 Plus |  | [41] |
| GSE41663 | Psoriatic lesional vs non-lesional skin | 81 | Affymetrix Human Genome U133 Plus |  | [41] |
| GSE50790 | Psoriatic lesional vs non-lesional skin | 8 | Affymetrix Human Genome U133 Plus |  | [42] |
| GSE53552 | Psoriatic lesional (with/without treatment) vs non-lesional skin | 99 | Affymetrix Human Genome U133 Plus |  | [43] |
| GSE54456 | Control vs Psoriasis | 174 | Illumina Genome Analyzer (Homo sapiens) | Validation of key driver subnetworks | ^[44-46]^ |
| **Methylome** | | | | | |
| GSE31835 | Control vs Psoriasis (involved and uninvolved) | 37 | Illumina Human Methylation27 array | 1. MSEA of methylome; 2. Meta-MSEA | [47] |
| GSE42634 | Control vs Psoriasis | 12 | NimbleGen Human DNA Methylation 385K Promoter Plus CpG Island Array |  | -- |
| GSE63315 | Control vs Psoriasis | 47 | Illumina Human Methylation450 array |  | [48] |
| **GWAS** | | | | | |
| phs000019.v1.p1 | Control vs Psoriasis | 2825 | Sequenom single base extension assays | 1. MSEA of GWAS; 2. Meta-MSEA | [9] |
| **eQTLs** | | | | | |
| Blood eQTLs | Blood cells | >20000 | 10 studies | SNP-to-gene mappings | ^[21, 23-32]^ |
| Skin eQTLs | Normal and Psoriatic skin | >300 | 3 studies |  | [25, 31, 32] |
| **Tissue-specific gene regulatory networks** | | | | | |
| GIANT Bayesian networks | Skin and blood tissues | >38000 | Bayesian methodology | Key Driver Analysis in gene regulatory networks | ^[37]^ |

**Note:** ^*^ The transcriptome and methylome were retrieved from GEO database (www.ncbi.nlm.nih.gov/geo/) and GWAS was retrieved from dbGAP ([www.ncbi.nlm.nih.gov/gap](http://www.ncbi.nlm.nih.gov/gap)).

**Supplementary Table S2. Knowledge-based artificial pathways from published GWAS studies and large-scale meta-profiling of diverse collections of gene expression data sets.**

| **Gene Sets** | **Size** | **Member Genes** | **References** |
| --- | --- | --- | --- |
| GWAS Positive | 119 | ADO, ADRA1B, AKAP13, ANXA6, B3GNT2, BHLHE40, BRAP, C1orf141, CAMK2G, CAPZB, CARD6, CARM1, CDKAL1, CFL1, CHUK, CLIC6, COG6, CTNNA3, DDX58, DEFB1, ELMO1, ERAP1, ETS1, EXOC2, FAM27L, FASLG, FBXL19, FIBP, FLJ16341, FOSL1, FUBP1, FUT2, HLA-B, HLA-C, IFIH1, IFNAR1, IFNLR1, IKBKE, IL12B, IL13, IL23A, IL23R, IL28RA, IL31, IL4, ILF3, IRF4, JAK2, KCNH7, KLF13, KLF4, KLLN, KLRC4, KLRK1, LCE3A, LCE3D, LINC00330, LOC144817, LRRC7, MAPKAPK5, MHC, NFKBIA, NFKBIZ, NOS2, OSTN, PAK7, PLCL2, POL1, POL3S, POU2F3, PRDX5, PRM3, PRSS53, PSMA6, PTEN, PTPN2, PTRF, QTRT1, REL, REV3L, RGS6, RN7SKP9, RNF114, RNF145, RP11-61O1.1, RPS26, RUNX1, RUNX3, RUSC2, SDC4, SLC45A1, SLC9A8, SNAI1, SNORD74, SOCS1, SPATA2, STARD6, STAT2, STAT3, STX1B, TNFAIP3, TNFRSF9, TNIP, TNIP1, TP63, TRAF3IP2, TRIM47, TRIM65, TSC1, TYK2, UBAC2, USP49, YDJC, ZC3H12C, ZDHHC23, ZMIZ1, ZNF313, ZNF365, ZNF816A | [5-15] |
| IL23/IL17 Immune | 106 | ACT1, AHR, AIRE, ANAPC5, BATF, BCL2, CAMP, CARD9, CCL5, CCL6, CCL20, CCL22, CCR6, CRP, CSF2, CSF3, CXCL1, CXCL2, CXCL5, DECTIN1, ELAVL1, PTGER1, PTGER3, PTGER4, FOXP3, GATA3, GSK3B, GZMA, GZMB, HIF1A, HSP90AA1, IFNA1, IFNA2, IFNG, IKBKE, IL10, IL12A, IL12B, IL12RB1, IL15, IL17A, IL17B, IL17C, IL17D, IL17F, IL17RA, IL17RB, IL17RC, IL17RD, IL18, IL1B, IL1R1, IL2, IL21R, IL22, IL23A, IL23R, IL25, IL27, IL2RG, IL4, IL6, IL7, IL8, IL9, IRF4, ITCH, ITGB3, JUN, LCN2, MAF, MAPK1, MAPK8, MYD88, NFKB1, NFKB2, NOD2, PRDM1, RORC, S100A7, S100A8, S100A9, SRSF1, STAT1, STAT3, STAT4, STAT6, STUB1, TAB2, TAB3, TAX1BP1, TBK1, TBX21, TGFB1, TGFB3, TNF, TNFAIP3, TRAF2, TRAF3, TRAF3IP2, TRAF4, TRAF5, TRAF6, TYK2, USP25, USP7 | [16, 17] |
| Gene Signatures | 527 | ACPP, ALOX12B, ARG1, ATP12A, ATP1B1, C10orf99, CCL20, CD24, CDH1, CDSN, CLCA2, CLEC3B, CRABP2, DEFB4A, DEFB4B, DSC2, EGR1, FGFBP1, GM2A, HAL, HBB, HPSE, ID1, IL19, IL1RN, IL36A, IL36G, IL8, KLK13, KLK6, KLK8, KYNU, LCE2C, LCE3D, LCN2, LTF, NAMPT, OASL, PDZK1IP1, PI3, PKP1, PLAT, PRSS3, RAB5A, RGS20, S100A12, S100A7A, S100A9, SERPINB13, SERPINB3, SERPINB4, SLC39A6, SLC6A14, SPRR1A, SPRR2B, SPRR2C, SPTLC2, SQLE, TCN1, TGM1, TGM3, TWF1, VNN3, WFDC12, WNT5A, CASP14, FOXE1, KRT25, KRT27, KRT71, STS, TNFRSF19, TP63, UGCG, ZDHHC21, ABCA12, NFKBIZ, PKP3, CNFN, CTTN, EHF, FGFR3, HBA1, HBA2, MBNL1, MBNL2, SCAMP1, NAV3, CHRNA9, CP, EDNRA, MALAT1, PTGER3, PTGR1, RGS5, SLC7A1, TTC37, IL36RN, TMPO, SLURP1, MCL1, VAV3, WTAP, CTNNA1, TREX2, CLCN3, NDRG2, NPR3, ANGPTL1, ARF6, CXADR, FAF1, FBXO45, GNG12, HYAL4, PTPN12, TMOD3, VSNL1, WASL, XDH, ZC3H12A, RORA, WNT16, SERPINB7, ZFP36L2, RHCG, SLC26A9, AKR1B10, HMGCS1, RAB27B, SPRR3, SGPP2, DHX9, DNAJC3, GDA, KLF3, KREMEN1, MPZL2, NME7, HSD17B1, HIPK1, LYNX1, SLC5A1, REEP1, JMY, ABCD3, TMC5, UGT1A3, ATP6V1A, COPA, HECTD1, HIPK3, PRSS27, TMPRSS11D, APOE, IL4R, LEP, SELE, DKK1, DSG3, FCGR3B, LYZ, PPARG, AURKA, GATA3, KRT19, CCL2, CFB, CFH, IL1B, AOC3, CD2, HBEGF, LCK, SELL, ADIPOQ, ADRB2, AGTR1, ANG, CCL22, CCL4, CCR7, CD48, CDH3, CFD, CXCL10, CXCL13, CXCL9, CXCR4, CYP7B1, EPHA2, F12, F3, GPC3, HMMR, IL7R, KLRB1, LPL, LYN, MMP1, MMP12, MMP7, NPY1R, PLN, RBP4, SLC7A5, SLPI, TGFBR3, TIMP3, TIMP4, TNFRSF21, PRKCQ, SOD2, HMOX1, STAT1, AURKB, BIRC5, CCNA2, CCNB1, CCNE1, CENPE, CLEC7A, CRAT, ESRRG, FABP4, HSD11B1, HSD17B2, KIF11, KRT6A, MXD1, MYLK, NR3C2, PCK1, RORC, RRM2, TOP2A, TTK, UPP1, XAF1, CXCR2, EPCAM, SCGB2A2, TPBG, IL37, CDK1, PNP, CTPS1, GBP1, HPGDS, ID4, IDO1, TYMP, FADS2, LRP4, BUB1B, PBK, RAB38, APOC1, CST6, KRT16, FUT2, GGH, NOD2, ADAMDEC1, AQP9, ARSF, BTC, C7, CA6, CCL18, CCL27, CCL8, CD3D, COCH, COL21A1, CXCL1, CXCL2, CYP2C18, CYP2J2, DHRS9, DNASE1L3, FADS1, FPR1, GABRP, GAL, GDPD3, GZMA, GZMB, HS3ST3A1, KCNJ15, KCNK5, KLK10, LGALS3BP, LIPG, LPHN3, LRP8, LRRC17, MSMB, MUC7, NETO2, OAS2, PCOLCE2, PGLYRP4, PIP, PLA2G2F, PLA2G3, PLSCR1, POSTN, RDH16, RGS1, RNASE4, SEMA3G, SERPINA3, SGCG, SLC16A6, SLC23A2, SLC7A11, SMPD3, SSPN, TMPRSS4, WIF1, PTTG1, S100A2, UBD, ACADL, ACSBG1, ACTC1, ACTG2, ADH1B, ALDH1A3, AMMECR1, ARNTL2, ATP6V1B1, BCAR3, BCL2A1, CALML3, CCNB2, CD207, CDC20, CDKN3, CDO1, CEP55, CIDEC, CKS2, CRY2, DDX58, EIF4EBP1, EMX2, FABP7, FHL1, FOSL1, FOXC1, GINS2, GLDC, GNA15, GPD1, GPRASP1, GPX2, GSTA3, HERC6, HK2, HMGCS2, HSD3B1, IFI44, IFIT3, INA, IRF8, ISG15, ISG20, IVL, KIAA0101, KIF20A, KIF4A, KPNA2, KRT15, KRT6B, KRT6C, MAD2L1, MB, MELK, MX1, MX2, MYH11, MYL9, NCAPG, NDC80, NMI, OAS1, PCP4, PDK4, PHLDA2, PLAC8, PLCB4, PPARGC1A, PPIF, PPP1R1A, PRDM1, PYCARD, RTP4, S100A8, S100P, SCO2, SERPINB1, SPC25, SULT2B1, TAGLN, TK1, TNMD, TPPP, TPX2, TRIM22, UBE2C, UCHL3, ZBTB16, ZWINT, APOD, APOL1, ATP10B, ATP6V0A4, CDHR1, CHI3L2, CLDN8, EPHX3, EREG, ERO1L, FAM189A2, FCGBP, FLVCR2, FUT3, FXYD1, FXYD6, GALNT6, IFI27, IFI6, IGFBP6, LAMP3, LSP1, LYVE1, MFAP5, NRN1, PAMR1, PLBD1, PLLP, RSAD2, SCGB1D2, SCGB2A1, TMEM47, TSPAN8, VNN1, IFIH1, ADAP2, AIM2, APOBEC3A, APOBEC3B, C12orf5, C14orf132, C1orf115, CARHSP1, CH25H, CHAC1, CHP2, CMAHP, CNN1, COBL, CORO2B, COTL1, COX7A1, CRCT1, CRIP1, CRYAB, DDX60, DLGAP5, EPN3, FAM134B, FAR2, FERMT1, FOXA1, GINS3, GLRX3, HAO2, HSPA4L, IDH3A, IFI44L, IFIT1, IRF7, ITM2A, KIF18B, KRT17, MICALL1, MPHOSPH6, MREG, NCALD, OAS3, OTUB2, PGBD5, PHYHIP, PLA2G16, PLIN1, POLE2, POLR3G, PRC1, PRR15L, PRSS53, RAI14, RAI2, RCAN2, RERGL, RHOBTB3, SAMD9, SAMSN1, SELENBP1, SERHL2, SFT2D2, SPRR1B, STIL, SYNM, TMEM255A, TMEM40, TNIP3, TNNI2, TRBC2, TTC39A, UBE2L6, ZBED2, ZNF135, ZNF273, ZSCAN18 | [18, 19] |

**Supplementary Tables S3 – S9 were provided in separate Excel sheets.**

**Supplementary Table S10. In silico mining of the KD genes using bioinformatics tools.**

| **Key Drivers** | **Mouse Phenotypes from Gene Knockouts** | **Gene Signatures** | **PolySearch**  **(PubMed>5)** | **COREMINE**  **(Sig<0.01)** | **GWAS**  **(p<5E-8)** | **EWAS**  **(p<1E-5)** |
| --- | --- | --- | --- | --- | --- | --- |
| **KDs for Common Supersets** | | | | | | |
| APP | Central nervous system (CNS) inflammation | N/A | N/A | N/A | N/A | **Yes** |
| BIRC3 | abnormal IL12 secretion; abnormal cytokine secretion | N/A | N/A | N/A | N/A | **Yes** |
| CD247 | N/A | N/A | N/A | N/A | N/A | **Yes** |
| CD3E | N/A | N/A | N/A | N/A | N/A | N/A |
| CD48 | N/A | **Yes** | N/A | N/A | N/A | **Yes** |
| CD74 | decreased IL2 secretion | N/A | N/A | N/A | N/A | N/A |
| CSF1R | abnormal circulating cytokine level | N/A | N/A | N/A | N/A | N/A |
| CTSH | N/A | N/A | N/A | N/A | N/A | N/A |
| FYB | decreased IL2 secretion | N/A | N/A | N/A | N/A | N/A |
| GRB2 | N/A | N/A | N/A | N/A | N/A | N/A |
| GZMA | N/A | **Yes** | N/A | N/A | N/A | N/A |
| HLA-A | CNS inflammation; abnormal cytokine level | N/A | N/A | **Yes** | **Yes** | N/A |
| HLA-C | N/A | N/A | N/A | **Yes** | **Yes** | N/A |
| HLA-DQB1 | decreased IL12 secretion; abnormal cytokine secretion | N/A | N/A | N/A | **Yes** | N/A |
| HLA-DRB1 | decreased IL12 secretion; abnormal cytokine secretion | N/A | N/A | **Yes** | **Yes** | N/A |
| HLA-E | N/A | N/A | N/A | N/A | **Yes** | N/A |
| HLA-F | N/A | N/A | N/A | N/A | **Yes** | N/A |
| HLA-G | N/A | N/A | N/A | N/A | **Yes** | N/A |
| ICAM1 | abnormal interleukin level; inflammation | N/A | **Yes** | **Yes** | N/A | N/A |
| IFITM2 | N/A | N/A | N/A | N/A | N/A | N/A |
| IL15 | Spontaneous skin ulceration; abnormal cytokine secretion | N/A | **Yes** | **Yes** | N/A | N/A |
| IL1B | Psoriasis | **Yes** | **Yes** | **Yes** | N/A | **Yes** |
| IL32 | N/A | N/A | **Yes** | N/A | N/A | N/A |
| LCK | decreased IL2 secretion | **Yes** | N/A | N/A | N/A | N/A |
| LCP1 | decreased IL1 secretion | N/A | N/A | N/A | N/A | **Yes** |
| LGALS9 | N/A | N/A | N/A | N/A | **Yes** | N/A |
| MYD88 | decreased inflammatory response; abnormal chemokine level | N/A | N/A | N/A | N/A | N/A |
| PSMC5 | N/A | N/A | N/A | N/A | N/A | N/A |
| PSMD6 | N/A | N/A | N/A | N/A | N/A | N/A |
| PTPN6 | Psoriasis | N/A | N/A | N/A | N/A | **Yes** |
| SRGN | N/A | N/A | N/A | N/A | N/A | **Yes** |
| STAT1 | decreased IL1 beta secretion; increased circulating IL12b level | **Yes** | **Yes** | N/A | N/A | N/A |
| STAT3 | Psoriasis | N/A | **Yes** | **Yes** | **Yes** | N/A |
| TNFAIP3 | increased inflammatory response; abnormal cytokine secretion | N/A | **Yes** | **Yes** | **Yes** | N/A |
| TNFSF10 | liver inflammation | N/A | N/A | N/A | N/A | **Yes** |
| TRAF1 | N/A | N/A | N/A | N/A | N/A | **Yes** |
| **KDs for GWAS/EWAS-unique Supersets** | | | | | | |
| ACAP2 | N/A | N/A | N/A | N/A | N/A | N/A |
| ALG8 | N/A | N/A | N/A | N/A | N/A | N/A |
| ARMCX6 | N/A | N/A | N/A | N/A | N/A | N/A |
| BPTF | N/A | N/A | N/A | N/A | N/A | N/A |
| CCDC12 | N/A | N/A | N/A | N/A | N/A | N/A |
| COQ5 | N/A | N/A | N/A | N/A | N/A | N/A |
| DYNLT1 | N/A | N/A | N/A | N/A | N/A | N/A |
| ENTPD5 | N/A | N/A | N/A | N/A | N/A | N/A |
| FPGT | N/A | N/A | N/A | N/A | N/A | N/A |
| GARS | N/A | N/A | N/A | N/A | N/A | N/A |
| MARS | N/A | N/A | N/A | N/A | N/A | N/A |
| MFF | N/A | N/A | N/A | N/A | N/A | N/A |
| PTCD3 | N/A | N/A | N/A | N/A | N/A | N/A |
| RAB25 | abnormal skin condition | N/A | N/A | N/A | N/A | N/A |
| RASA2 | N/A | N/A | N/A | N/A | N/A | N/A |
| STK36 | N/A | N/A | N/A | N/A | N/A | N/A |
| TMEM40 | N/A | **Yes** | N/A | N/A | N/A | N/A |
| YARS | N/A | N/A | N/A | N/A | N/A | N/A |
| ZNF226 | N/A | N/A | N/A | N/A | N/A | N/A |
| COL4A2 | N/A | N/A | N/A | N/A | N/A | **Yes** |
| G0S2 | N/A | N/A | N/A | N/A | N/A | N/A |
| GHR | decreased susceptibility to autoimmune diabetes | N/A | N/A | N/A | N/A | N/A |
| KRT18 | N/A | N/A | N/A | N/A | N/A | N/A |
| PARC | N/A | N/A | N/A | N/A | N/A | N/A |
| PRKCZ | immune system phenotype | N/A | N/A | N/A | N/A | N/A |
| RBA1 | N/A | N/A | N/A | N/A | N/A | N/A |

**Supplementary Table S11. Gene expression perturbations of key driver subnetwork in lesional skin tissue in psoriasis patients.**

| **KD Subnetworks** | **Network Size** | **Significant Member Genes in Enrichment Analysis ^*^** |
| --- | --- | --- |
| HLA-G | 19 | CCL5, CFLAR, **IFITM1**, RELB, IFIH1, PSMB8, **LGALS3BP**, PARP12, TAP1, **HMOX1**, **OASL**, TAP2 |
| STAT1 | 20 | TNFAIP2, BST2, UBE2D3, TXN, IFITM1, **ISG15**, STAT2, CASP1, WARS, TNFSF10, **NMI**, STAT3, TAP1, **OASL**, **STAT1** |
| TNFAIP3 | 44 | BHLHE40, IL32, MPZL1, YWHAZ, LAPTM5, IFITM1, TRAF1, TNIP2, CD47, ZFP36, WARS, IER2, TNFSF10, **UCHL3**, CD83, **NMI**, UGCG, CASP4, **CXCR4**, **UBE2L6**, **KYNU** |
| BIRC3 | 35 | TDRD7, BST2, CLINT1, IFNGR2, TRAF2, SLA, NFKB2, SP100, MAP3K5, IFITM1, TRAF1, RELB, **IFIT3**, BIRC3, IFNGR1, WARS, JUNB, IER2, **IL7R**, SAT1, TNFSF10, **ISG20**, IRF1, UGCG, **LYN**, SGK1 |
| TNFSF10 | 12 | TNFRSF10B, CD47, TNFRSF10A, TNFSF10, **TRIM22**, **NMI**, TRIM14, **UBE2L6** |
| HLA-E | 51 | SP100, TAPBP, IFITM1, IFI35, PSME1, CD47, IGFBP3, STAT2, **IFIT3**, PML, IFNGR1, PLEK, **MX1**, **PLSCR1**, LAP3, **ISG20**, **OAS2**, PARP12, STAT3, IFI16, TAP1, **HMOX1**, **OASL**, **SPTLC2** |
| HLA-A | 37 | HLA-G, IL15, HLA-C, HLA-A, HLA-F, HLA-E, KRT5, B2M, TXN, SP100, TAPBP, IFITM1, HLA-B, GSTO1, **ISG15**, RAC2, WARS, SP110, SERPINB9, PARP12, IFI16, TAP1, **HMOX1**, **IFI27**, **STAT1** |
| IL1B | 13 | PELI1, PTGS2, SERPINE1, **IL1B**, IL6, PLAUR, MCL1, **CCL2**, **CCL20** |
| LCK | 11 | NLRC3, ZAP70, PTPRC, SYK, RAC2, CD247, SPOCK2, **LCK**, SH2D2A |
| IL15 | 72 | SP100, CFLAR, PRDX1, YWHAZ, ARHGDIB, LAPTM5, IFITM1, PSMB9, PLAUR, PLAU, PSME1, SRGN, PTP4A1, RAC2, LDHA, WARS, **MX1**, **PLSCR1**, SAT1, TNFSF10, PSMB8, **LGALS3BP**, IRF1, **OAS2**, **SOD2**, CASP7, SQRDL, STAT3, CASP4, MYD88, **STAT1**, **UBE2L6** |

**Note: ^*^** The transcriptomic patterns of the KD subnetworks were assessed between 92 psoriatic and 82 normal punch biopsies using GSEA. The nominal p value estimates the statistical significance of the enrichment score for a single KD subnetwork. The FDR is the estimated probability that a KD subnetwork with a given normalized enrichment score represents a false positive finding. The genes in bold are consistent with the gene signatures in Supplementary Table S4.

References

1. Song WM, Zhang B: **Multiscale Embedded Gene Co-expression Network Analysis.** *Plos Computational Biology* 2015, **11**.

2. Joshi-Tope G, Gillespie M, Vastrik I, D'Eustachio P, Schmidt E, de Bono B, Jassal B, Gopinath GR, Wu GR, Matthews L, et al: **Reactome: a knowledgebase of biological pathways.** *Nucleic Acids Research* 2005, **33:**D428-D432.

3. Ogata H, Goto S, Sato K, Fujibuchi W, Bono H, Kanehisa M: **KEGG: Kyoto Encyclopedia of Genes and Genomes.** *Nucleic Acids Research* 1999, **27:**29-34.

4. Hindorff LA, Sethupathy P, Junkins HA, Ramos EM, Mehta JP, Collins FS, Manolio TA: **Potential etiologic and functional implications of genome-wide association loci for human diseases and traits.** *Proc Natl Acad Sci U S A* 2009, **106:**9362-9367.

5. Baurecht H, Hotze M, Brand S, Buning C, Cormican P, Corvin A, Ellinghaus D, Ellinghaus E, Esparza-Gordillo J, Folster-Holst R, et al: **Genome-wide comparative analysis of atopic dermatitis and psoriasis gives insight into opposing genetic mechanisms.** *American Journal of Human Genetics* 2015, **96:**104-120.

6. Capon F, Bijlmakers MJ, Wolf N, Quaranta M, Huffmeier U, Allen M, Timms K, Abkevich V, Gutin A, Smith R, et al: **Identification of ZNF313/RNF114 as a novel psoriasis susceptibility gene.** *Hum Mol Genet* 2008, **17:**1938-1945.

7. Ellinghaus E, Ellinghaus D, Stuart PE, Nair RP, Debrus S, Raelson JV, Belouchi M, Fournier H, Reinhard C, Ding J, et al: **Genome-wide association study identifies a psoriasis susceptibility locus at TRAF3IP2.** *Nat Genet* 2010, **42:**991-995.

8. Liu Y, Helms C, Liao W, Zaba LC, Duan S, Gardner J, Wise C, Miner A, Malloy MJ, Pullinger CR, et al: **A genome-wide association study of psoriasis and psoriatic arthritis identifies new disease loci.** *Plos Genetics* 2008, **4:**e1000041.

9. Nair RP, Duffin KC, Helms C, Ding J, Stuart PE, Goldgar D, Gudjonsson JE, Li Y, Tejasvi T, Feng BJ, et al: **Genome-wide scan reveals association of psoriasis with IL-23 and NF-kappaB pathways.** *Nat Genet* 2009, **41:**199-204.

10. Strange A, Capon F, Spencer CC, Knight J, Weale ME, Allen MH, Barton A, Band G, Bellenguez C, Bergboer JG, et al: **A genome-wide association study identifies new psoriasis susceptibility loci and an interaction between HLA-C and ERAP1.** *Nat Genet* 2010, **42:**985-990.

11. Stuart PE, Nair RP, Ellinghaus E, Ding J, Tejasvi T, Gudjonsson JE, Li Y, Weidinger S, Eberlein B, Gieger C, et al: **Genome-wide association analysis identifies three psoriasis susceptibility loci.** *Nat Genet* 2010, **42:**1000-1004.

12. Tsoi LC, Spain SL, Ellinghaus E, Stuart PE, Capon F, Knight J, Tejasvi T, Kang HM, Allen MH, Lambert S, et al: **Enhanced meta-analysis and replication studies identify five new psoriasis susceptibility loci.** *Nat Commun* 2015, **6:**7001.

13. Tsoi LC, Stuart PE, Tian C, Gudjonsson JE, Das S, Zawistowski M, Ellinghaus E, Barker JN, Chandran V, Dand N, et al: **Large scale meta-analysis characterizes genetic architecture for common psoriasis associated variants.** *Nat Commun* 2017, **8:**15382.

14. Yin X, Low HQ, Wang L, Li Y, Ellinghaus E, Han J, Estivill X, Sun L, Zuo X, Shen C, et al: **Genome-wide meta-analysis identifies multiple novel associations and ethnic heterogeneity of psoriasis susceptibility.** *Nat Commun* 2015, **6:**6916.

15. Zhang XJ, Huang W, Yang S, Sun LD, Zhang FY, Zhu QX, Zhang FR, Zhang C, Du WH, Pu XM, et al: **Psoriasis genome-wide association study identifies susceptibility variants within LCE gene cluster at 1q21.** *Nat Genet* 2009, **41:**205-210.

16. Di Cesare A, Di Meglio P, Nestle FO: **The IL-23/Th17 Axis in the Immunopathogenesis of Psoriasis.** *Journal of Investigative Dermatology* 2009, **129:**1339-1350.

17. Lubberts E: **The IL-23-IL-17 axis in inflammatory arthritis.** *Nature Reviews Rheumatology* 2015, **11:**415-429.

18. Inkeles MS, Scumpia PO, Swindell WR, Lopez D, Teles RMB, Graeber TG, Meller S, Homey B, Elder JT, Gilliet M, et al: **Comparison of Molecular Signatures from Multiple Skin Diseases Identifies Mechanisms of Immunopathogenesis.** *Journal of Investigative Dermatology* 2015, **135:**151-159.

19. Qu XYA, Freudenberg JM, Sanseau P, Rajpal DK: **Integrative clinical transcriptomics analyses for new therapeutic intervention strategies: a psoriasis case study.** *Drug Discovery Today* 2014, **19:**1364-1371.

20. Thornton T, McPeek MS: **Case-control association testing with related individuals: A more powerful quasi-likelihood score test.** *American Journal of Human Genetics* 2007, **81:**321-337.

21. Emilsson V, Thorleifsson G, Zhang B, Leonardson AS, Zink F, Zhu J, Carlson S, Helgason A, Walters GB, Gunnarsdottir S, et al: **Genetics of gene expression and its effect on disease.** *Nature* 2008, **452:**423-U422.

22. Greenawalt DM, Dobrin R, Chudin E, Hatoum IJ, Suver C, Beaulaurier J, Zhang B, Castro V, Zhu J, Sieberts SK, et al: **A survey of the genetics of stomach, liver, and adipose gene expression from a morbidly obese cohort.** *Genome Research* 2011, **21:**1008-1016.

23. Dixon AL, Liang L, Moffatt MF, Chen W, Heath S, Wong KC, Taylor J, Burnett E, Gut I, Farrall M, et al: **A genome-wide association study of global gene expression.** *Nature genetics* 2007, **39:**1202-1207.

24. Fehrmann RS, Jansen RC, Veldink JH, Westra HJ, Arends D, Bonder MJ, Fu J, Deelen P, Groen HJ, Smolonska A, et al: **Trans-eQTLs reveal that independent genetic variants associated with a complex phenotype converge on intermediate genes, with a major role for the HLA.** *Plos Genetics* 2011, **7:**e1002197.

25. Nica AC, Parts L, Glass D, Nisbet J, Barrett A, Sekowska M, Travers M, Potter S, Grundberg E, Small K, et al: **The architecture of gene regulatory variation across multiple human tissues: the MuTHER study.** *Plos Genetics* 2011, **7:**e1002003.

26. Montgomery SB, Sammeth M, Gutierrez-Arcelus M, Lach RP, Ingle C, Nisbett J, Guigo R, Dermitzakis ET: **Transcriptome genetics using second generation sequencing in a Caucasian population.** *Nature* 2010, **464:**773-U151.

27. Stranger BE, Montgomery SB, Dimas AS, Parts L, Stegle O, Ingle CE, Sekowska M, Smith GD, Evans D, Gutierrez-Arcelus M, et al: **Patterns of Cis Regulatory Variation in Diverse Human Populations.** *PLoS genetics* 2012, **8:**272-284.

28. Stranger BE, Nica AC, Forrest MS, Dimas A, Bird CP, Beazley C, Ingle CE, Dunning M, Flicek P, Koller D, et al: **Population genomics of human gene expression.** *Nature genetics* 2007, **39:**1217-1224.

29. Dimas AS, Deutsch S, Stranger BE, Montgomery SB, Borel C, Attar-Cohen H, Ingle C, Beazley C, Arcelus MG, Sekowska M, et al: **Common Regulatory Variation Impacts Gene Expression in a Cell Type-Dependent Manner.** *Science* 2009, **325:**1246-1250.

30. Duan S, Huang RS, Zhang W, Bleibel WK, Roe CA, Clark TA, Chen TX, Schweitzer AC, Blume JE, Cox NJ, Dolan ME: **Genetic architecture of transcript-level variation in humans.** *American Journal of Human Genetics* 2008, **82:**1101-1113.

31. Ding J, Gudjonsson JE, Liang LM, Stuart PE, Li Y, Chen W, Weichenthal M, Ellinghaus E, Franke A, Cookson W, et al: **Gene Expression in Skin and Lymphoblastoid Cells: Refined Statistical Method Reveals Extensive Overlap in cis-eQTL Signals.** *Am J Hum Genet* 2010, **87:**779-789.

32. Ardlie KG, DeLuca DS, Segre AV, Sullivan TJ, Young TR, Gelfand ET, Trowbridge CA, Maller JB, Tukiainen T, Lek M, et al: **The Genotype-Tissue Expression (GTEx) pilot analysis: Multitissue gene regulation in humans.** *Science* 2015, **348:**648-660.

33. Boyle AP, Hong EL, Hariharan M, Cheng Y, Schaub MA, Kasowski M, Karczewski KJ, Park J, Hitz BC, Weng S, et al: **Annotation of functional variation in personal genomes using RegulomeDB.** *Genome Research* 2012, **22:**1790-1797.

34. Maher B: **ENCODE: The human encyclopaedia.** *Nature* 2012, **489:**46-48.

35. Shu L, Zhao Y, Kurt Z, Byars SG, Tukiainen T, Kettunen J, Orozco LD, Pellegrini M, Lusis AJ, Ripatti S, et al: **Mergeomics: multidimensional data integration to identify pathogenic perturbations to biological systems.** *BMC genomics* 2016, **17:**874.

36. Shu L, Zhao Y, Kurt Z, Byars SG, Tukiainen T, Kettunen J, Ripatti S, Zhang B, Inouye M, Makinen V-P: **Mergeomics: integration of diverse genomics resources to identify pathogenic perturbations to biological systems.** *bioRxiv* 2016**:**036012.

37. Greene CS, Krishnan A, Wong AK, Ricciotti E, Zelaya RA, Himmelstein DS, Zhang R, Hartmann BM, Zaslavsky E, Sealfon SC, et al: **Understanding multicellular function and disease with human tissue-specific networks.** *Nat Genet* 2015, **47:**569-576.

38. Swindell WR, Johnston A, Carbajal S, Han G, Wohn C, Lu J, Xing X, Nair RP, Voorhees JJ, Elder JT, et al: **Genome-wide expression profiling of five mouse models identifies similarities and differences with human psoriasis.** *PLoS One* 2011, **6:**e18266.

39. Yao Y, Richman L, Morehouse C, de los Reyes M, Higgs BW, Boutrin A, White B, Coyle A, Krueger J, Kiener PA, Jallal B: **Type I interferon: potential therapeutic target for psoriasis?** *PLoS One* 2008, **3:**e2737.

40. Suarez-Farinas M, Li K, Fuentes-Duculan J, Hayden K, Brodmerkel C, Krueger JG: **Expanding the psoriasis disease profile: interrogation of the skin and serum of patients with moderate-to-severe psoriasis.** *J Invest Dermatol* 2012, **132:**2552-2564.

41. Bigler J, Rand HA, Kerkof K, Timour M, Russell CB: **Cross-study homogeneity of psoriasis gene expression in skin across a large expression range.** *PLoS One* 2013, **8:**e52242.

42. Swindell WR, Xing X, Stuart PE, Chen CS, Aphale A, Nair RP, Voorhees JJ, Elder JT, Johnston A, Gudjonsson JE: **Heterogeneity of inflammatory and cytokine networks in chronic plaque psoriasis.** *PLoS One* 2012, **7:**e34594.

43. Russell CB, Rand H, Bigler J, Kerkof K, Timour M, Bautista E, Krueger JG, Salinger DH, Welcher AA, Martin DA: **Gene expression profiles normalized in psoriatic skin by treatment with brodalumab, a human anti-IL-17 receptor monoclonal antibody.** *J Immunol* 2014, **192:**3828-3836.

44. Elder JT, Tsoi LC, Iyer MK, Stuart PE, Swindell WR, Gudjonsson JE, Tejasvi T, Sarkar MK, Li B, Ding J, et al: **Analysis of long non-coding RNAs highlights tissue-specific expression patterns and epigenetic profiles in normal and psoriatic skin.** *Journal of Investigative Dermatology* 2015, **135:**S75-S75.

45. Tsoi LC, Iyer MK, Stuart PE, Swindell WR, Gudjonsson JE, Tejasvi T, Sarkar MK, Li BS, Ding J, Voorhees JJ, et al: **Analysis of long non-coding RNAs highlights tissue-specific expression patterns and epigenetic profiles in normal and psoriatic skin.** *Genome Biol* 2015, **16**.

46. Liang Y, Tsoi LC, Xing XY, Beamer MA, Swindell WR, Sarkar MK, Berthier CC, Stuart PE, Harms PW, Nair RP, et al: **A gene network regulated by the transcription factor VGLL3 as a promoter of sex-biased autoimmune diseases.** *Nature Immunology* 2017, **18:**152-160.

47. Roberson EDO, Liu Y, Ryan C, Joyce CE, Duan SH, Cao L, Martin A, Liao W, Menter A, Bowcock AM: **A Subset of Methylated CpG Sites Differentiate Psoriatic from Normal Skin.** *Journal of Investigative Dermatology* 2012, **132:**583-592.

48. Gu X, Nylander E, Coates PJ, Fahraeus R, Nylander K: **Correlation between Reversal of DNA Methylation and Clinical Symptoms in Psoriatic Epidermis Following Narrow-Band UVB Phototherapy.** *J Invest Dermatol* 2015, **135:**2077-2083.
